# Integrated metabolite analysis and health-relevant *in vitro* functionality of white, red, and orange maize (*Zea mays* L.) from the Peruvian Andean race *Cabanita* at different maturity stages

**DOI:** 10.3389/fnut.2023.1132228

**Published:** 2023-02-28

**Authors:** Lena Gálvez Ranilla, Gastón Zolla, Ana Afaray-Carazas, Miguel Vera-Vega, Hugo Huanuqueño, Huber Begazo-Gutiérrez, Rosana Chirinos, Romina Pedreschi, Kalidas Shetty

**Affiliations:** ^1^Laboratory of Research in Food Science, Universidad Catolica de Santa Maria, Arequipa, Perú; ^2^Escuela Profesional de Ingeniería de Industria Alimentaria, Departamento de Ciencias e Ingenierías Biológicas y Químicas, Facultad de Ciencias e Ingenierías Biológicas y Químicas, Universidad Catolica de Santa Maria, Arequipa, Perú; ^3^Laboratorio de Fisiología Molecular de Plantas, PIPS de Cereales y Granos Nativos, Facultad de Agronomía, Universidad Nacional Agraria La Molina, Lima, Perú; ^4^Programa de Investigación y Proyección Social en Maíz, Facultad de Agronomía, Universidad Nacional Agraria La Molina, Lima, Perú; ^5^Estación Experimental Agraria Arequipa, Instituto Nacional de Innovación Agraria (INIA), Arequipa, Perú; ^6^Instituto de Biotecnología, Universidad Nacional Agraria La Molina, Lima, Perú; ^7^Escuela de Agronomía, Facultad de Ciencias Agronómicas y de los Alimentos, Pontificia Universidad Católica de Valparaíso, Valparaíso, Chile; ^8^Millennium Institute Center for Genome Regulation (CRG), Santiago, Chile; ^9^Department of Plant Sciences, North Dakota State University, Fargo, ND, United States

**Keywords:** *Zea mays*, Peruvian maize, *Cabanita*, primary metabolites, secondary metabolites, antioxidant capacity, hyperglycemia, biodiversity

## Abstract

The high maize (*Zea mays L.*) diversity in Peru has been recognized worldwide, but the investigation focused on its integral health-relevant and bioactive characterization is limited. Therefore, this research aimed at studying the variability of the primary and the secondary (free and dietary fiber-bound phenolic, and carotenoid compounds) metabolites of three maize types (white, red, and orange) from the Peruvian Andean race *Cabanita* at different maturity stages (milk-S1, dough-S2, and mature-S3) using targeted and untargeted methods. In addition, their antioxidant potential, and α-amylase and α-glucosidase inhibitory activities relevant for hyperglycemia management were investigated using *in vitro* models. Results revealed a high effect of the maize type and the maturity stage. All maize types had hydroxybenzoic and hydroxycinnamic acids in their free phenolic fractions, whereas major bound phenolic compounds were ferulic acid, ferulic acid derivatives, and *p*-coumaric acid. Flavonoids such as luteolin derivatives and anthocyanins were specific in the orange and red maize, respectively. The orange and red groups showed higher phenolic ranges (free + bound) (223.9–274.4 mg/100 g DW, 193.4– 229.8 mg/100 g DW for the orange and red maize, respectively) than the white maize (162.2–225.0 mg/100 g DW). Xanthophylls (lutein, zeaxanthin, neoxanthin, and a lutein isomer) were detected in all maize types. However, the orange maize showed the highest total carotenoid contents (3.19–5.87 μg/g DW). Most phenolic and carotenoid compounds decreased with kernel maturity in all cases. In relation to the primary metabolites, all maize types had similar fatty acid contents (linoleic acid > oleic acid > palmitic acid > α-linolenic acid > stearic acid) which increased with kernel development. Simple sugars, alcohols, amino acids, free fatty acids, organic acids, amines, and phytosterols declined along with grain maturity and were overall more abundant in white maize at S1. The *in vitro* functionality was similar among *Cabanita* maize types, but it decreased with the grain development, and showed a high correlation with the hydrophilic free phenolic fraction. Current results suggest that the nutraceutical characteristics of orange and white *Cabanita* maize are better at S1 and S2 stages while the red maize would be more beneficial at S3.

## 1. Introduction

Maize (*Zea mays* L. ssp. *mays*) originated about 9,000 years ago in Mexico, and Latin America is considered the center of its genetic diversity and primary domestication (1–3). This cereal is staple food in Mesoamerican and Latin American countries since it is the base of many traditional preparations. It has been reported that the conservation and sustainable use of Latin American maize landrace diversity is fundamental for worldwide food security (1). Hence, efforts at multiple levels should be focused on the characterization of the genetically heterogenous landrace material as the base for further breeding improvements relevant for food security and health among indigenous food systems (1, 4).

The diversity of maize landrace populations is represented in races, which are identified according to their common botanical characteristics, geographical distribution, ecological adaptation, and cultural importance (uses and customs) (5–7). Mexico and Peru have concentrated around 30 percent of the Latin American maize diversity including 59 and 52 races, respectively (7, 8). The Peruvian Andean region with its great variety of ecological features has the highest maize phenotypic diversity worldwide (7, 9, 10). However, limited scientific information exist about Andean maize diversity which is compromising its adequate conservation and essential health relevant uses.

Whole cereal grains are valuable sources of carbohydrates, proteins, dietary fiber, minerals, and vitamins along with other critical bioactive metabolites with known health-promoting benefits (11, 12). The regular intake of whole grains has been inversely correlated with lower incidence of several chronic non-communicable diseases including type 2 diabetes (13), cardiovascular disease (14), and some types of cancer (15, 16). Maize contains nutritionally relevant macro and micronutrients mainly carbohydrates, lipids (with mono and polyunsaturated fatty acids), vitamins, minerals, and resistant starch (17). In addition, biologically active functional compounds such as phenolic compounds, carotenoids, tocopherols, and phytosterols have been reported in maize (17). In fact, unique phenolic and carotenoid profiles have been reported in different maize landraces linked to variable nutraceutical properties (18). Accordingly, more studies are needed to fully characterize maize landraces, targeting those that are the base of needs of food security and economy in many geographical areas such as the Andean region.

The maize race *Cabanita* has been cultivated since the Pre-Inca period in the southern Andean region of Arequipa in Peru at around 3,000 meters of altitude (19). Ears of *Cabanita* race have a conic-cylindrical shape and exhibit variable kernel pigmentations with predominance of white and partially red colored-pericarps (19). In a previous study, some Peruvian maize races including *Arequipeño*, *Cabanita*, *Kculli*, *Granada*, and *Coruca* races were evaluated in relation to their phenolic composition, *in vitro* anti-hyperglycemia, and anti-obesity potential (20). *Cabanita* kernels showed the second highest total oxygen radical absorbance antioxidant capacity (ORAC) and hyperglycemia management-relevant α-amylase inhibition following the purple-colored maize group (*Kculli* race) (20). More recently, *Cabanita* maize from two different provinces in Arequipa (Peru) were evaluated in relation to their physical characteristics, bioactive (phenolic and carotenoid) composition and *in vitro* antioxidant capacity (19). Although *Cabanita* samples from both provinces showed a certain grade of similarity according to the multivariate PCA (Principal Component Analysis), in general maize cultivated under Andean environments with naturally higher ecological stress factors such as higher altitudes and lower temperatures showed higher phenolic and antioxidant capacity ranges (19).

The intake of this traditional Andean maize is mostly in the mature dried form. Andean farmers still maintain the postharvest traditional practices along the *Cabanita* maize production chain. Once the maize ears have reached their highest length and a certain moisture level which is subjectively measured based on the farmer’s experience, the plants are cut and dried in a piled form in the same land. Thereafter, dried plants are transported to the farmer’s warehouses where maize ears are unshelled and exposed directly to the sun until complete drying (19). Dried grains are then consumed roasted, or further milled and used as flour in different culinary preparations.

Several studies focused mostly on sweet and waxy maize improved varieties have shown that bioactive compounds such as phenolic antioxidants and carotenoids vary depending on the kernel maturation stage. Phenolic compounds such as anthocyanins from different Asian colored waxy maize genotypes increased along the kernel maturation from 20 to 35 days after pollination (DAP) (21). Similarly, Zhang et al. (22) observed an increase of the total phenolic contents (TPC) in mature kernels from a yellow maize variety (48 DAP). The increase of carotenoids such as lutein, zeaxanthin, *α*-cryptoxanthin, *β*-cryptoxanthin has also been reported during the kernel maturation of some sweet maize varieties from China (23). On the contrary, the total phenolic and total carotenoids contents decreased at the end of grain maturation in yellow maize bred in United States (116 DAS, days after seeding) (24). These discrepancies reveal that different factors including genetic factors (variety), the time of harvest, and the agroecological conditions of maize cultivation may influence the bioactive composition during the maize kernel maturation. Consequently, the research on this topic should be performed case by case, according to specific ecological environments.

As a second stage follow up studies of previous advances to characterize the Peruvian Andean maize *Cabanita* (19), the objective of current research was to study the primary (polar compounds and fatty acids) and secondary metabolite composition (free and bound phenolics and carotenoid compounds), and the *in vitro* health-relevant functional properties of three selected *Cabanita* types (white, red, and orange pigmented kernels) harvested at different maturity stages, using targeted and untargeted metabolomic platforms. The *in vitro* model based functionality of *Cabanita* maize was evaluated in relation to its antioxidant potential and inhibitory activity against key digestive enzymes (α-amylase and α-glucosidase) relevant for hyperglycemia modulation. Results from this study will contribute with important biochemical and metabolomic information for the characterization, and conservation of the maize race *Cabanita*. In addition, information from this research would be important to diversify the consumption options of this Andean maize beyond the traditional mature form. This would likely lead to potential beneficial effects of food crops at health and economical levels among indigenous communities in the future.

## 2. Materials and methods

### 2.1. Cultivation of *Cabanita* maize and sampling

The germplasm of *Cabanita* maize (*Zea mays* L.) collected in a previous study was used (19). Maize with sample codes CAW, CCR, COM representing white, red, and orange kernels were selected for current study considering the pigmentation diversity found in *Cabanita* maize race (19). These maize samples were obtained from the province of Caylloma (Cabanaconde district) located in the southern Andean region of Arequipa in Peru and were stored under refrigeration (2–5°C) (19). The field experiment was performed in the nursery garden *Santa Maria* at the *Universidad Catolica de Santa Maria* located in the Sachaca district (S: 16° 41′ 93.9″; W: 071° 56′ 34.0″; 2,240 meters of altitude), province of Arequipa (Arequipa, Peru).

*Cabanita* maize was cultivated in 20 L pots under open air and sun light exposure from 25 November 2020 to 28 June 2021. Commercial prepared soil (containing humus, field soil, and manure) was used and its physico-chemical characteristics are shown in Supplementary Table S1. The meteorological conditions during the maize plant development until sample harvest are shown in Supplementary Table S2. Groups of 6 pots were sown in three consecutive weeks (total 18 pots per maize type) and 5 *Cabanita* seeds were sown in each pot (sowing dates: 25 November, 2 December, and 9 December 2020). This procedure was applied to ensure the number of biological replicates (four) at three maturity stages per type of maize (white, red, orange) for the current study. The group of ears harvested from a single pot was considered a biological replicate (from 1 to 4 ears were obtained per pot). Additional supplementation with commercial fertilizers (urea and NPK + micronutrients) was performed during the vegetative period of maize plants (from week 2 to 11 after sowing) and the phytosanitary control was undertaken using conventional practices for the cultivation of maize in combination with the use of ecological insect traps. Well water was used for the irrigation which was carried out under field capacity in a similar way as in field cultivation.

During the plant reproductive stage, the female inflorescences were promptly protected with a plastic bag until the emergence of the styles (silks). The pollination was manually developed using composite pollen collected from mature tassels (male inflorescence) of plants from the same maize type. Once pollinated, each ear was protected with paper bags until physiological maturity. This procedure avoided the cross-pollination among different maize types. Ears were collected at three different grain maturity stages according to the grain physical appearance and moisture contents (25, 26). The milky stage (S1) is characterized by the starch accumulation and the observation of a milky white fluid upon finger pressure (25). In the dough stage (S2), the grain is still soft and humid, with intermediate humidity, whereas the physiological mature stage (S3) corresponds to the completion of kernel development, and a black layer is formed at the base of the kernel (25, 26). In case of the white maize type, S1 corresponded to 28 DAP and 79% moisture, S2 to 39 DAP and 68% moisture, and S3 to 75 DAP and 45% moisture. For the red type maize, S1 was at 33 DAP and 74% moisture, S2 at 36 DAP and 68% moisture, and S3 was at 77 DAP and 45% moisture. In the orange type, S1 corresponded to 32 DAP and 75% moisture, S2 to 43 DAP and 64% moisture, and S3 to 76 DAP and 46% moisture.

After harvest, ear samples were immediately stored under refrigeration (5–8°C) and transported to the laboratory. Husks were eliminated, and samples (ear and kernels) were evaluated in relation to their physical characteristics as will be described in next section. Kernels were separated, pooled per biological replicate, and frozen (−20°C). This process was developed within the 24 h after harvest. Afterwards, samples were freeze-dried in a FreeZone benchtop freeze dryer (Labconco, Kansas, MO, United States) for 60 h, at –40°C, and 0.008 mbar of vacuum pressure. Then, dried kernels were milled in a A11 Basic analytical mill (IKA, Germany) with liquid nitrogen to a powdered flour, packed in 50 ml polypropylene tubes protected from light, and stored at –20°C until analysis.

### 2.2. Enzymes and reagents

Baker yeast α-glucosidase (EC 3.2.1.20), and porcine pancreas α-amylase (EC 3.2.1.1) were from Sigma-Aldrich (St. Louis, MO, United States). Phenolic standards (gallic acid, vanillic acid, caffeic acid, ferulic acid, *p*-coumaric acid, cyanidin chloride, and quercetin aglycone), carotenoid standards (lutein, zeaxanthin, *β*-cryptoxanthin), and the Folin–Ciocalteu reagent were from Sigma-Aldrich. The (±)-6-hydroxy-2,5,7,8-tetramethyl-chromane-2-carboxilic acid (Trolox), and the 2,2-diphenyl-1-picrylhydrazyl (DPPH˙), and 2–2′-azino-bis(3ethylbenothiazoline-6-sulfonic acid) (ABTS^·+^) radicals were purchased from Sigma-Aldrich. Pyridine, phenyl-β-d-glucopyranoside, methoxyamine hydrochloride, *N,O*-bis(trimethylsilyl)trifluoroacetamide (BSTFA), and methyl undecanoate were from Sigma-Aldrich.

### 2.3. Physical measurements and moisture determination

Relevant physical descriptors were evaluated in fresh harvested ears and kernels (per maturity stage and maize type) per replicate according to the International Board for Plant Genetic Resources (27). The weight (g), length (cm), and central diameter (cm) were measured in ears whereas the length (mm), width (mm), and thickness (mm) were determined in kernels. The kernel moisture was monitored periodically to characterize the maturity stage for harvest and was determined by a gravimetric method at 105°C (28).

### 2.4. Extraction of phenolic and carotenoid compounds from maize samples

#### 2.4.1. Free and bound phenolic fractions

The extraction of phenolic compounds from the lyophilized maize samples were performed according to Ranilla et al. (29) with some modifications. An amount of 1 g of maize sample was mixed with 4 ml of 0.1% HCl methanol/acetone/water (45, 45, 10, v/v/v) for the extraction of the free phenolic fraction. The bound phenolic compounds were released from the insoluble free-phenolic residue by alkaline hydrolysis with 3 N NaOH following same procedure as Ranilla et al. (29). Final extracts were reconstituted in milliQ water and stored at-20°C until analysis.

#### 2.4.2. Carotenoids

The procedure of Fuentes-Cardenas et al. (19) was followed. A saponification process was first applied with 80% KOH (w/v) and methanol:ethyl acetate (6, 4, v/v) solvent was used for the carotenoid extraction until a clear final extract was obtained. Carotenoids were extracted under light and oxygen protection and analyzed by ultra high-performance liquid chromatography (UHPLC) after the extraction process the same day.

### 2.5. Targeted metabolomic analysis

#### 2.5.1. Analysis of phenolic compounds by ultra high-performance liquid chromatography

Free and bound phenolic extracts were filtered using a polyvinylidene difluoride filters (PVDF, 0.22 μm) and the separation was carried out in a Kinetex C18 reverse-phase analytical column (100 × 2.1 mm i.d., 1.7 μm) with a Kinetex C18 guard column (5 × 2.1 mm i.d., 1.7 μm) (Phenomenex Inc., Torrance, CA, United States). The injection volume was 5 μl and samples were injected at 0.2 ml/min flow rate in an Ultimate 3,000 RS UHPLC system (Thermo Fisher Scientific, Waltham, MA, United States) with a diode array detector, a quaternary pump, an autosampler, and column oven. Acetonitrile and 0.1% formic acid in water were used as mobile phases and the same gradient and chromatographic parameters reported by Ranilla et al. (29) and Vargas-Yana et al. (30) were applied. Eluates were monitored from 200 to 600 nm. The Chromeleon SR4 software version 7.2 (Thermo Fisher Scientific) was used for chromatograms and data processing. The identification of phenolic compounds was based on their retention times and ultraviolet–visible spectra characteristics compared with those of the library data and external standards. Calibration curves with external standards were used for the quantification of phenolic compounds (*r*^2^ ≥ 0.9990). Hydroxybenzoic phenolic acids (HBA) (unidentified 1 and 2) were quantified at 280 nm and expressed as vanillic acid. Vanillic acid derivatives (with similar UV–VIS spectra as that of vanillic acid but with different retention times) were expressed also as vanillic acid. Hydroxycinnamic phenolic acids (HCA) including ferulic, *p*-coumaric, and caffeic acid derivatives were quantified at 320 nm and expressed as ferulic, *p*-coumaric, and caffeic acids, respectively. Flavonoids such as luteolin derivatives (with similar UV–VIS spectra as that of luteolin, but with different retention times), and anthocyanins were detected at 360 and 525 nm, and quantified using quercetin aglycone and cyanidin chloride external standards, respectively. All results were expressed as mg per 100 g DW (dried weight).

#### 2.5.2. Analysis of carotenoid compounds by UHPLC

The analysis of carotenoid compounds was performed with a YMC carotenoid C30 reverse-phase analytical column (150 × 4.6 mm i.d., 3 μm) coupled to a YMC C30 guard column (10 × 4.0 mm, 3 μm) (YMC CO., LTD, Japan) using the same UHPLC system as previously described for the phenolic compound analyses. Filtered carotenoid extracts (0.22 μl, PVDF filter) were injected at 1.7 ml/min flow rate and monitored at 450 nm. A ternary gradient elution was used (methanol, dichloromethane, acetonitrile) and same reverse-phase chromatographic conditions as those reported by Fuentes-Cardenas et al. (19) were applied. The retention time and UV–VIS spectra characteristics of external carotenoid standards and the library data were used for the identification of carotenoid compounds in evaluated samples. In addition, the information of carotenoid analyses in other maize samples from reported literature was also useful for the identification of carotenoid isomers. Calibration curves made with external standards were used for the quantification of carotenoids (*r*^2^ ≥ 0.9900) and results were presented as μg per g sample DW. Lutein and zeaxanthin compounds and their isomers were quantified as lutein and zeaxanthin, respectively. Unidentified carotenoid compounds, neoxanthin, and violaxanthin isomers were expressed as lutein. *β*-cryptoxanthin isomers were expressed as *β*-cryptoxanthin.

#### 2.5.3. Analysis of the total phenolic contents

The TPC in the free and bound phenolic extracts were evaluated according to Singleton and Rossi (31) using the Folin–Ciocalteu method. Results were presented as mg of gallic acid equivalents (GAE) per 100 g DW.

#### 2.5.4. Fatty acids profiles by gas-chromatography with flame ionization detector

The analysis was adapted from Uarrota et al. (32). The fatty acid methyl ester synthesis (FAME) was obtained by combining ~55 mg of lyophilized maize sample with 70 μl 10 N KOH (prepared in HPLC water) and 530 μl of HPLC grade methanol in a reaction tube. The mix was incubated in a water bath at 55–60°C for 1.5 h with periodic agitation every 30 min. Tubes were then cooled down to room temperature and drops of fuming H_2_SO_4_ (24 N) were carefully added. An incubation step was repeated as previously described. Samples were cooled, then 500 μl of hexane and 10 μl of internal standard (methyl undecanoate, 26.16 mg/ml) were added. The tubes were vortexed for 2 min and centrifuged at 17,000 *g* for 10 min at 4°C. The upper layer was transferred to a vial with an insert and 1 μl was injected in an Agilent 7890B gas chromatography system coupled to a flame ionization detector (FID) (Agilent Technologies, Santa Clara, CA, United States). A 2560 capillary gas-chromatography (GC) column (100 m × 250 μm × 0.2 μm) (Supelco, Bellefonte, PA, United States). The injector temperature was set at 220°C, the FID detector at 225°C, air flow (400 ml/min), hydrogen flow (35 ml/min), helium flow (1.6 ml/min), using an injection with a split ratio of 50:1. The chromatographic run was set up at 80°C (initial temperature) and increased to 225°C with a heating ramp at a rate of 25°C per min, and held for 25 min. The retention times of detected peaks from samples were compared with those of external standards for fatty acid identification. Calibration curves (*r*^2^ ≥ 0.9900) with palmitic, stearic, oleic, linoleic, and *α*-linolenic acids were used for the quantification of fatty acids in maize samples and results are presented as mg per g sample DW.

### 2.6. Untargeted metabolomic analysis of polar compounds by gas chromatography mass spectrometry

The extraction of polar metabolites from maize samples, the derivatization process, and the instrumental parameters for the gas chromatography mass spectrometry (GC–MS) analysis were the same as reported by Fuentealba et al. (33). An Agilent 7890B gas chromatography system equipped with a 5977A single quadrupole MS, a PAL3 autosampler, an electron impact ionization source was used (Agilent Technologies). A HP-5 ms Ultra Inert column (30 m × 0.25 mm × 0.25 μm) (Agilent) was used for the separation of polar compounds. The Mass Hunter Quantitative software (Agilent Technologies) was used for the deconvolution and data processing. For peak identification, their retention times and mass spectra were compared with data from NIST14 and a home library (obtained with commercial standards). Results are shown as the relative response of each compound calculated considering their respective sample weight, an internal standard (phenyl-β-d-glucopyranoside), and a quality control (QC) composite sample from all maize samples (33).

### 2.7. *In vitro* functionality of *Cabanita* maize samples

#### 2.7.1. Extraction of hydrophilic and lipophilic fractions

The soluble hydrophilic and lipophilic fractions from lyophilized maize samples were considered for the *in vitro* assays. The hydrophilic and lipophilic fractions were extracted with 80% methanol and dichloromethane; respectively, following same extraction parameters described by Fuentes-Cardenas et al. (19).

#### 2.7.2. Antioxidant capacity by the 2,2-diphenyl-1-picrylhydrazyl radical scavenging assay

The method of Duarte-Almeida et al. (34) adapted to a microplate reader (Biotek Synergy HTX, Agilent Technologies) with modifications reported by Fuentes-Cardenas et al. (19) was applied. Results are shown as μmol Trolox equivalents per 100 g DW using Trolox calibration curves prepared in methanol (20–160 μM), and dichloromethane (10–120 μM) for the hydrophilic and lipophilic fractions, respectively.

#### 2.7.3. Antioxidant capacity by the 2.2′-azino-bis (3-ethylbenzothiazoline-6-sulfonic acid) radical cation (ABTS^·+^) scavenging assay

The hydrophilic and lipophilic extracts were evaluated according to Fuentealba et al. (35) using a Biotek Synergy HTX microplate reader (Agilent Technologies). Results were expressed as μmol Trolox equivalents per 100 g DW based on calibration curves built with Trolox standard in methanol and dichloromethane for the evaluation of hydrophilic and lipophilic fractions, respectively. The Trolox concentration ranges used in calibration curves were the same as those shown for the DPPH method.

#### 2.7.4. α-Glucosidase and α-amylase inhibitory activity

The hydrophilic and lipophilic fractions used for the determination of the α-amylase inhibitory activity were obtained similarly as Fuentes-Cardenas et al. (19) but using a different sample and solvent ratio for the extraction (0.5 g sample in 12.5 ml 80% methanol). Final extracts (hydrophilic and lipophilic) were vacuum-evaporated to dryness at 45°C and reconstituted in 2 ml 0.02 M NaPO_4_ buffer (pH 6.9) (35). In case of the α-glucosidase inhibition analysis, same extraction conditions were assayed as Fuentes-Cardenas et al. (19), and final hydrophilic and lipophilic extracts were also vacuum-evaporated to dryness but resuspended in 1 ml of 0.1 M KPO_4_ buffer (pH 6.9) (35). The inhibitory activity against α-amylase and α-glucosidase enzymes were determined with the same methodology reported by Gonzalez-Muñoz et al. (36). The percentage (%) of inhibition at different sample amounts was reported.

### 2.8. Statistical analysis

Results (from four independent biological replicates) were expressed as means ± standard deviation. A two-way analysis of variance (ANOVA) with the LSD test were carried out to determine significant differences between the means (*p* < 0.05) using the software Infostat^1^ (accessed from October to November, 2022). Pearson correlations among all data were explored using the Statgraphics Centurion XVI software (StatPoint Inc., Rockville, MD, United States). All data (from the targeted and untargeted metabolite analyses, the physical characteristics, and the functionality assays) were evaluated through the multivariate principal component analysis (PCA) using the Metaboanalyst software version 5.0^2^ (accessed on 2 October, 2022). For the PCA, data were first mean-centered and divided by the standard deviation of each variable. Afterward, an ANOVA with the Tukey’s HSD *post hoc* analysis (*p* < 0.01) was carried out to identify significant variables. The heat map or cluster analysis was performed using the Euclidean distance and the Ward algorithm in Metaboanalyst with the top significant metabolites or variables (*p* < 0.01).

## 3. Results and discussion

### 3.1. Physical changes of *Cabanita* maize types at different maturity stages

The maturity stages of *Cabanita* maize characterized by the DAP and moisture levels were similar among evaluated maize types. However, their vegetative periods (from the plant emergence stage to the end of the tasseling time) were somewhat different (26). This period occurred at around 9, 10, and 11 weeks after the sowing stage in case of the orange, white, and red maize types, respectively. Consequently, the start of the reproductive period (emergence of the silk or the female inflorescence) was earlier in the orange maize type (13 weeks after sowing), followed by the white (15 weeks after sowing), and the red maize (16 weeks after sowing). This may be important for the adequate planning of maize cultivation periods since Andean farmers traditionally sow maize mixing different *Cabanita* types in the same land.

**Figure 1:**
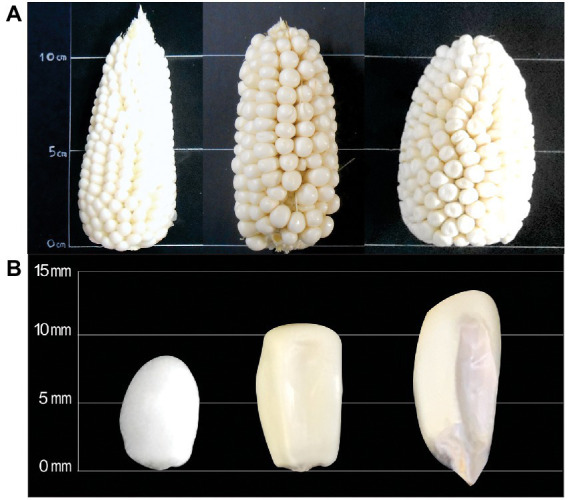
Changes of ear **(A)** and kernel **(B)** physical characteristics of white *Cabanita* maize at different maturity stages (S1, S2, S3, from left to right).

**Figure 2:**
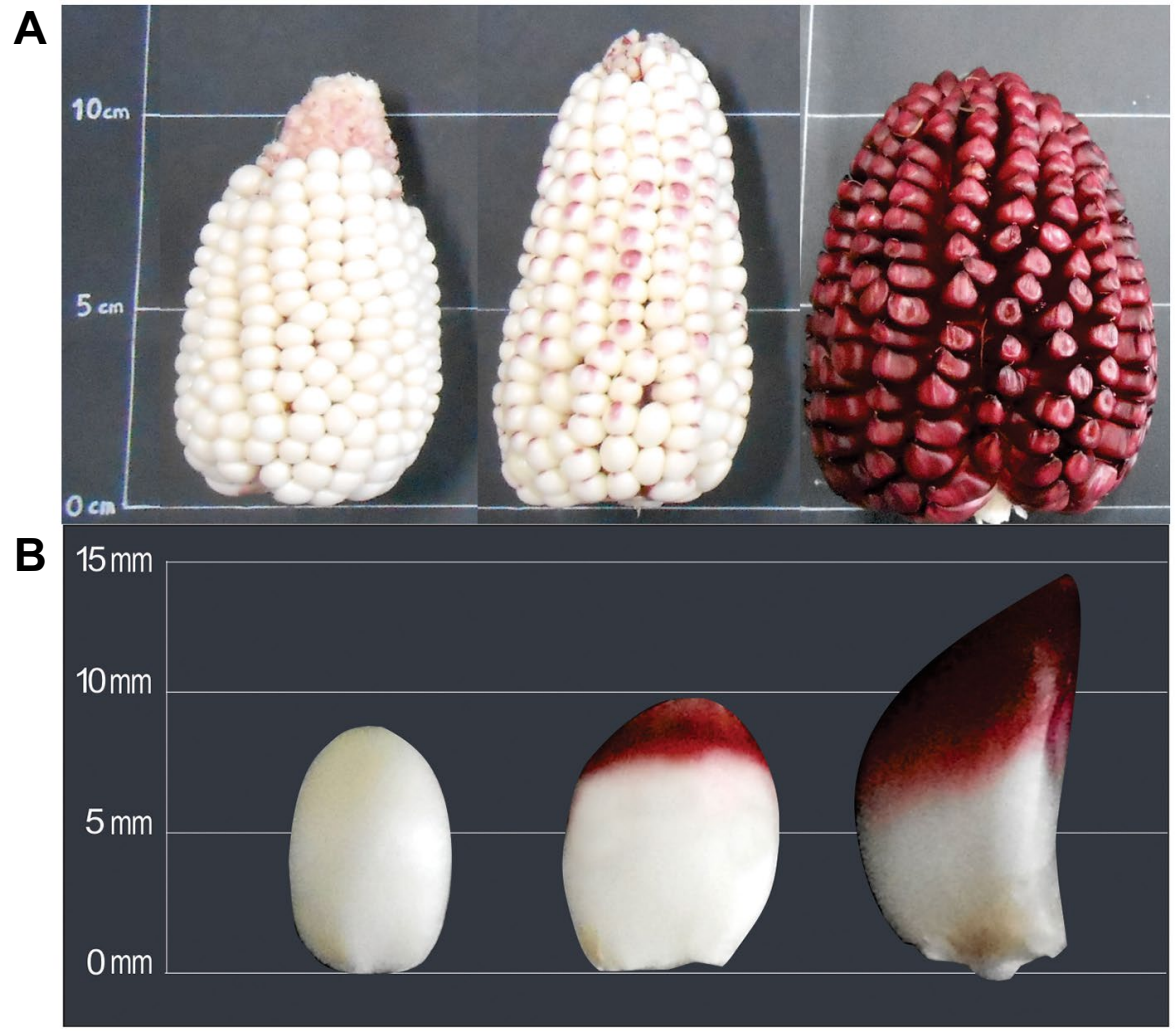
Changes of ear **(A)** and kernel **(B)** physical characteristics of red *Cabanita* maize at different maturity stages (S1, S2, S3, from left to right).

**Figure 3:**
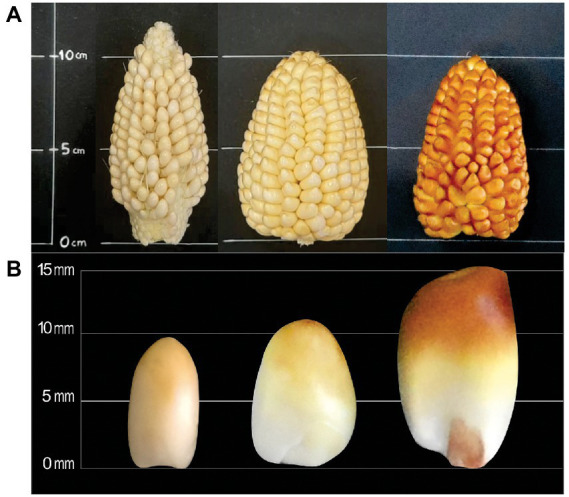
Changes of ear **(A)** and kernel **(B)** physical characteristics of orange *Cabanita* maize at different maturity stages (S1, S2, S3, from left to right).

The physical changes of ears and kernels from the three types of *Cabanita* maize along the maturity stages are shown in Figures 1–3. The development of all maize types was characterized by changes in the pericarp color. The white maize type varied from light-white to white-yellow at S3 which may be related to the accumulation of dry matter with maturity (Figure 1) (37). In case of the red type, the pigmentation appeared in the S2 stage as a small spot at the stigma-end of the kernel that then extended toward almost the half of the grain at S3 (Figure 2). Hong et al. (38) observed a similar trend in a purple-pericarp sweet corn; however, the purple pigment fully spread until the base of the kernel at the highest maturity phase (32 DAP). The orange maize varied from light-yellow at S1 to orange at S3 and this pigmentation only reached the middle of the kernels similarly as in the red case.

Table 1 shows the physical characteristics evaluated in kernels sampled at different developmental stages. No significant interaction between the maize type (M) and the maturity stage (S) factors was found in any of the measured physical parameters. The variation of the ear weight and diameter, and the kernel width along the maturation period was similar in all maize types. Nevertheless, the ear length, kernel length, and thickness were influenced by the maize type. The ear length was higher in the white and red maize than in the orange type, but this latter showed higher kernel length ranges than the former. The maturity stage factor (S) was significant in all the physical characteristics except the ear length which remained almost similar during the maturation. The ear weight, and diameter along with the kernel length, width, and thickness increased with maturation. Overall, the yield-relevant physical parameter (ear weight) was similar among all maize classes; however, some morphological differences have been observed among the kernel types (Figures 1–3). Fuentes-Cardenas et al. (19) also studied the Peruvian maize race *Cabanita* and reported no differences in the quantitative physical characteristics between the CAW (white), CCR (red), and COM (orange) maize samples (which are the parental seeds of the white, red, and orange maize types evaluated in the current study). However, mature, and dried ears and kernels were evaluated in such study.

**Table 1:** Physical characteristics of *Cabanita* maize kernels at different pigmentations and maturity stages.

Maize type	Stage	Ear (cm)			Kernel (mm)		
		Weight	Length	Diameter	Length	Width	Thickness
White	S1	70.4 ± 19.9c	9.1 ± 0.7ab	4.2 ± 0.4f	0.80 ± 0.04e	0.79 ± 0.07d	0.59 ± 0.04e
	S2	150.0 ± 72.9a	9.9 ± 1.2a	5.3 ± 0.2abcd	1.11 ± 0.06 cd	0.80 ± 0.05 cd	0.61 ± 0.02de
	S3	138.4 ± 51.7ab	9.6 ± 0.7ab	5.6 ± 0.7abc	1.44 ± 0.10b	0.97 ± 0.10ab	0.71 ± 0.10abcd
Red	S1	88.8 ± 21.5bc	8.5 ± 0.9abc	4.6 ± 0.5def	0.95 ± 0.15de	0.83 ± 0.17bcd	0.63 ± 0.08cde
	S2	106.9 ± 36.6abc	9.9 ± 1.8a	5.1 ± 0.3 cde	0.93 ± 0.12e	0.89 ± 0.11abcd	0.74 ± 0.08ab
	S3	144.1 ± 64.6ab	9.3 ± 0.4ab	5.9 ± 0.8a	1.43 ± 0.15b	0.95 ± 0.14abc	0.73 ± 0.07ab
Orange	S1	77.0 ± 22.8c	8.1 ± 1.2bc	4.5 ± 0.6ef	1.19 ± 0.06c	0.82 ± 0.06bcd	0.66 ± 0.04bcde
	S2	76.0 ± 16.8c	7.4 ± 0.9c	5.1 ± 0.3bcde	1.21 ± 0.10c	0.82 ± 0.07 cd	0.72 ± 0.01abc
	S3	121.1 ± 18.3abc	8.6 ± 0.7abc	5.8 ± 0.5ab	1.65 ± 0.19a	1.03 ± 0.11a	0.77 ± 0.10a
*F* value	Maize (M)	1.49^ns^	7.23^**^	0.16^ns^	17.32^****^	0.46^ns^	4.40^*^
	Stage (S)	5.75^**^	0.99^ns^	19.68^****^	66.24^****^	9.29^***^	7.75^**^
	M × S	1.09^ns^	1.42^ns^	0.50^ns^	2.32^ns^	0.60^ns^	0.66^ns^

Different letters in the same column indicate significant statistical differences (*p < 0.05; **p < 0.01; ***p < 0.001; and ****p < 0.0001); ns, no significant.

### 3.2. Phenolic contents and profiles of *Cabanita* maize types at different maturity stages

The phenolic profiles and contents determined in the free and bound phenolic fractions of *Cabanita* maize samples are shown in Table 2. In case of the free phenolic fraction, all maize samples contained phenolic acids such as hydroxybenzoic (HBA) and hydroxycinnamic acids (HCA), but specific flavonoid types such as anthocyanin and luteolin derivatives were detected only in red and orange maize types, respectively.

**Table 2:** Phenolic profiles and contents by UHPLC (mg/100 g DW) in *Cabanita* maize kernels of different pigmentations and maturity stages.

Fraction	Compound	White			Red			Orange			*F*-value		
		S1	S2	S3	S1	S2	S3	S1	S2	S3	Maize (M)	Stage (S)	M × S
Free	HBA-1	30.6 ± 11.2a	16.6 ± 2.6c	2.8 ± 1.0d	23.2 ± 4.7abc	22.2 ± 1.6bc	2.5 ± 1.8d	27.6 ± 9.5ab	17.4 ± 1.3c	5.9 ± 1.8d	0.10^ns^	58.83^****^	1.78^ns^
	HBA-2	0.06 ± 0.05a	ND^1^	ND	ND	ND	ND	0.11 ± 0.08a	ND	ND			
	Vanillic acid derivatives^2^	13.6 ± 1.4a	5.6 ± 1.2c	2.0 ± 1.1d	10.8 ± 3.5ab	9.6 ± 1.2b	2.5 ± 0.5 cd	11.4 ± 4.8ab	5.2 ± 1.2c	2.5 ± 0.5 cd	0.98^ns^	58.05^****^	2.88^*^
	*Total HBA*	44.2 ± 11.6a	22.2 ± 3.1c	4.8 ± 2.0d	34.1 ± 7.8b	31.8 ± 1.9bc	5.1 ± 2.2d	39.2 ± 14.2ab	22.6 ± 2.1c	8.4 ± 1.8d	0.01^ns^	69.29^****^	2.47^*^
	*p*-Coumaric acid derivatives^3^	0.8 ± 0.3 cd	0.6 ± 0.2 cd	2.2 ± 0.9a	0.4 ± 0.1 cd	0.3 ± 0.1d	1.1 ± 0.4bc	0.6 ± 0.2 cd	0.53 ± 0.04 cd	1.6 ± 0.8ab	5.39^*^	22.38^****^	1.02^ns^
	Ferulic acid derivatives^4^	3.3 ± 0.5a	1.4 ± 0.2c	1.7 ± 0.5c	2.5 ± 0.5b	2.8 ± 0.5ab	1.7 ± 0.8c	2.5 ± 0.5b	1.2 ± 0.3c	1.7 ± 0.4c	3.96^*^	18.79^****^	6.44^***^
	Caffeic acid derivatives^5^	ND	0.2 ± 0.1bc	1.2 ± 0.6a	ND	ND	1.5 ± 0.6a	0.03 ± 0.00bc	0.02 ± 0.00c	0.6 ± 0.6Bb			
	*Total HCA*	4.1 ± 0.8abc	2.1 ± 0.2de	5.1 ± 1.3a	3.0 ± 0.6 cde	3.1 ± 0.5bcd	4.3 ± 1.0ab	3.1 ± 0.6bcd	1.7 ± 0.3e	3.8 ± 1.7bc	3.07^ns^	17.15^****^	1.78^ns^
	Luteolin derivatives^6^	ND	ND	ND	ND	ND	ND	22.7 ± 12.5a	11.3 ± 4.9ab	5.1 ± 4.4b			
	Total anthocyanins^7^	ND	ND	ND	0.6 ± 0.2a	1.4 ± 1.3a	14.5 ± 18.7a	ND	ND	ND			
	*Total flavonoids*	ND	ND	ND	0.6 ± 0.2b	1.4 ± 1.3b	14.5 ± 18.7ab	22.7 ± 12.5a	11.3 ± 4.9ab	5.1 ± 4.4b			
	Total UHPLC free	48.3 ± 11.6b	24.3 ± 3.2 cde	10.0 ± 3.3f	37.6 ± 8.2bc	36.2 ± 3.5bcd	23.8 ± 17.7de	65.0 ± 14.1a	35.6 ± 4.8bcd	17.3 ± 3.9ef	4.80^*^	38.22^****^	4.11^*^
	Free – TPC^8^	57.9 ± 17.3ab	27.0 ± 3.2c	32.3 ± 3.3c	38.0 ± 6.5bc	44.6 ± 6.5abc	53.6 ± 26.1ab	53.0 ± 11.4ab	37.6 ± 6.2bc	58.7 ± 23.5a	1.72^ns^	3.13^ns^	2.93^*^
Bound	*p*-Coumaric acid	4.9 ± 1.2bc	5.2 ± 1.1bc	7.5 ± 1.9ab	6.4 ± 4.3abc	4.1 ± 1.1c	9.4 ± 2.6a	5.9 ± 0.5bc	7.8 ± 2.4ab	7.2 ± 2.2abc	0.77^ns^	4.28^*^	1.98^ns^
	Ferulic acid	163.3 ± 7.4abc	150.1 ± 14.9bc	133.2 ± 21.2c	161.9 ± 42.8abc	142.5 ± 17.7c	177.2 ± 12.0ab	190.3 ± 11.8a	179.3 ± 23.5ab	177.5 ± 29.2ab	6.84^**^	1.29^ns^	1.64^ns^
	Ferulic acid derivatives^4^	8.5 ± 0.7d	10.8 ± 0.5d	11.6 ± 6.0d	12.0 ± 3.0 cd	10.6 ± 4.1d	19.4 ± 2.4ab	13.1 ± 1.2 cd	16.7 ± 6.2bc	22.0 ± 1.9a	11.92^***^	11.08^***^	1.75^ns^
	Total UHPLC bound	176.7 ± 7.9abc	166.1 ± 16.4bc	152.3 ± 28.4c	180.3 ± 49.8abc	157.1 ± 21.9c	206.0 ± 12.4a	209.4 ± 13.2a	203.8 ± 29.4ab	206.6 ± 29.7a	7.68^**^	0.97^ns^	1.72^ns^
	Bound – TPC^8^	155.8 ± 4.5bc	144.3 ± 10.1c	142.2 ± 38.0c	146.0 ± 23.4c	122.6 ± 35.1c	187.0 ± 7.0ab	181.3 ± 14.2ab	188.1 ± 29.9ab	197.1 ± 21.8a	11.23^***^	3.12^ns^	2.72^ns^
Total (free + bound)	UHPLC TPC	225.0 ± 9.2bcd	190.4 ± 14.2de	162.2 ± 29.2e	217.9 ± 44.4bcd	193.4 ± 21.3cde	229.8 ± 22.4bc	274.4 ± 26.8a	239.4 ± 27.4ab	223.9 ± 26.8bcd	12.45^***^	6.11^**^	2.70^ns^
	TPC^8^	213.7 ± 18.8bcd	171.4 ± 8.7e	174.5 ± 35.8de	184.0 ± 19.5cde	167.2 ± 37.6e	240.7 ± 29.1ab	234.3 ± 23.2ab	225.7 ± 32.1abc	255.8 ± 39.4a	10.90^***^	4.67^*^	3.14^*^

Different letters in the same row indicate significant statistical differences (*p < 0.05; **p < 0.01; ***p < 0.001; and ****p < 0.0001); ns, no significant. *F* values were calculated only in complete dataset per variable. HBA, hydroxybenzoic acids (HBA 1 and 2 indicate different unidentified HBA with different UV–VIS spectra). HCA, hydroxycinnamic acids. S1, S2, S3 indicate maturity stages. ^1^Non-detected. ^2^Expressed as vanillic acid. ^3^Expressed as *p*-coumaric acid. ^4^Expressed as ferulic acid. ^5^Expressed as caffeic acid. ^6^Expressed as quercetin aglycon. ^7^Expressed as cyanidin chloride. ^8^Folin–Ciocalteu TPC expressed as mg GAE/100 g DW.

For the HBA group, the contents were more influenced by the maturity stage (S) than by the maize type (M). The interaction of both factors (M × S) was significant on the vanillic acid derivatives and the total HBA contents. The highest total HBA contents were observed at stage S1, and white and orange maize had higher levels than red maize (44.2, 39.2, and 34.1 mg/100 g DW, for the white, orange, and red maize, respectively). With the kernel maturation, the total HBA concentrations decreased around 80–90% in all cases (from S1 to S3). At least 3 classes of HBA have been detected in all *Cabanita* samples (Supplementary Figures S1–S3). Major HBA at S1 was HBA-1 (λmax = 279 nm), followed by vanillic acid derivatives (λmax = 249, 289 nm). Contents of both HBA then decreased with maturity to reach similar concentrations at S3. A minor HBA compound (HBA-2) was only found at S1 in white and orange maize types. Giordano et al. (39) reported that the free vanillic acid levels found in open-pollinated maize varieties from Italy with variable kernel pigmentations decreased from 1.8–15 to 0–0.08 mg/100 g DW when maturation stages varied from 5 to 76 DAS, respectively. Similarly, the contents of vanillic and protocatechuic acids significantly decreased or disappeared with the grain development of waxy maize from 86–109 to 110–138 DAS (37). Besides vanillic acid, syringic and *p*-hydroxybenzoic acids have been also reported in maize (40, 41). Total free HBA ranges from current study (4.8–44.2 mg/100 g DW) were comparable to levels found in US yellow and Indian specialty maize kernels (~33.7 and 2.7–38 mg/100 g DW, respectively) (40, 41). Other cereals such as barley, wheat, and oat have shown lower free HBA concentrations (~15.5, 12.5, and 4.6 mg/100 g, respectively) (40).

A variable trend was observed in case of the HCA group (Table 2 and Supplementary Figures S4–S6). All *Cabanita* maize types contained *p*-coumaric, and ferulic acid derivatives whereas caffeic acid derivatives were detected at some maturity stages. These HCA derivatives may be soluble conjugated phenolic acids such as hydroxycinnamic acid amides (HCAAs) as was previously reported in different cereals (42, 43). Several HCAAs derived mostly from *p*-coumaric, ferulic and caffeic acids (*N,N*-di-*p*-coumaroylspermine, *N-p*-coumaroyl-*N*-feruloylputrescine, caffeoylputrescine) have been previously reported in the free phenolic fraction of maize from different origins (44, 45). Hence, further studies are necessary to better identify the HCA derivatives found in current research. The maturity (S) and maize type (M) showed an important effect on *p*-coumaric and ferulic acid derivatives, but the interaction of both factors was significant only on the ferulic acid derivatives contents. *p*-Coumaric acid and caffeic acid derivatives increased with kernel development. The increase of *p*-coumaric acid derivatives levels from S1 to S3 was on average 2.6-fold, and white and orange maize types exhibited higher ranges than the red maize (0.8–2.2, 0.6–1.6, and 0.4–1.1 mg/100 g DW for white, orange, and red maize, respectively). Conversely, the concentrations of ferulic acid derivatives declined by 32–48% from S1 to S3 in all maize groups. Different studies have shown variable tendencies of the HCA compounds with kernel maturity. The free ferulic and chlorogenic acid contents reduced with kernel maturation in several Italian maize varieties, and the same trend was observed in Chinese waxy pigmented maize samples with ferulic and *p*-coumaric acids (37, 39). Recently, Hu et al. (46) observed an overall increment of ferulic and *p*-coumaric acids during kernel maturation of sweet maize from China, whereas chlorogenic acid declined (from 15 to 30 DAP) after an initial increase (from 10 to 15 DAP). In the current study, the total HCA contents first decreased from S1 to S2 in white and orange maize types, then increased at S3 in all cases. The origin, maize type (genetic factors), and the harvesting time may explain differences found in this study.

Anthocyanins were present only in red maize, showing an increase from 0.6 mg/100 g DW at S1 to 14.5 mg/100 g DW by the end of kernel maturity. Other flavonoids such as luteolin derivatives were specific for orange maize samples and significantly decreased by 80% from S1 to S3 (from 22.7 to 5.1 mg/100 g DW). No flavonoids were detected in white *Cabanita*. Hong et al. (38) observed a continuous anthocyanin accumulation from 105 mg/100 g DW at 20 DAP to 179 mg/100 g DW at 36 DAP in purple-pericarp “supersweet” sweet maize. The increase of the total monomeric anthocyanin contents with kernel ripening has been also confirmed by different studies (21, 37, 47). The flavone luteolin has been reported in Indian Himalayan pigmented maize accessions and some Chinese maize hybrids (48, 49). In addition, a *C*-glycosylflavone known as maysin (a luteolin derivative) has also been found in mature maize seeds (49). The luteolin derivatives found in the current study (λmax = 256, 270, 349 nm) may be maysin or similar compounds that should be confirmed in future studies. However, higher concentrations of these flavones were found in the orange *Cabanita* maize at all maturity stages compared to levels obtained by Zhang et al. (49) (1.13 ng/g DW of maysin in mature seeds). *C*-glycosylflavones have shown potential neuroprotective properties relevant for Alzheimer’s disease prevention (50, 51).

The total free phenolic fraction decreased from S1 to S3, and its composition was variable depending on the kernel stage and maize type. HBA were the most important compounds in white and red maize at S1 and S2. In case of the orange group, HBA and luteolin derivatives highly contributed to the total free phenolic fraction at S1 and S2. This maize showed the highest total free phenolic contents at S1 among all samples (65 mg/100 g DW). The red maize was rich in HBA at S1, and S2 whereas anthocyanins were the major contributors to the free phenolic fraction at S3.

Major compound in the bound phenolic fraction was ferulic acid, followed by ferulic acid derivatives, and *p*-coumaric acid (Supplementary Figures S7–S9). The M × S interaction was not significant for any of the bound phenolic compounds; however, the maize type showed an important effect on the ferulic acid, ferulic acid derivatives, and the total bound UHPLC phenolic contents (Table 2). Orange and red maize types had higher ranges of ferulic acid (177.5–190.3 mg/100 g DW, 142.5–177.0 mg/100 g DW, and 133.2–163.3 mg/100 g DW, for the orange, red, and white maize, respectively), and ferulic acid derivatives than the white group (13.1–22.0 mg/100 g DW, 10.6–19.4 mg/100 g DW, and 8.5–11.6 mg/100 g DW, for the orange, red, and white maize, respectively). Consequently, higher total bound phenolic levels determined by UHPLC were found in orange and red maize, specially at S3 than in the white type. Kernel maturity highly influenced the *p*-coumaric acid and ferulic acid derivatives contents. Both compounds showed an increase of around 1.2–1.7-fold from S1 to S3. Ferulic acid remained almost stable from S1 to S3 in white and orange maize samples, and a similar trend was observed in their total UHPLC bound phenolic contents. In case of the red maize, ferulic acid and the total bound phenolic compounds first decreased from S1 to S2, to further increase at S3. Similar results as those obtained for white and orange *Cabanita* maize have been reported by Zhang et al. (22) in yellow maize. In that study, the total bound phenolic contents were stable with kernel maturation from 15 to 48 DAP (22). Nonetheless, the levels of bound ferulic and *p*-coumaric acids significantly reduced with kernel development (from 5 to 76 DAS) in several Italian maize samples whereas in other research same bound HCA showed a variable tendency depending on the genotype (39, 46).

On the whole, these results suggest differences in the metabolism of phenolic compounds during kernel development among the three types of *Cabanita* maize. A possible metabolic flux of precursors of hydroxybenzoic acids such as some intermediates of the shikimate or the phenylpropanoid pathways toward the biosynthesis of HCA derivatives may occur in case of the white and orange grains (52). HCA may be used as precursors for the biosynthesis of anthocyanins in case of the red maize. Enzymes involved in the biosynthesis of cell wall-relevant phenolic compounds may have been upregulated toward the flavone pathway in the orange maize explaining its overall higher ranges of bound phenolic compounds through the kernel growing process (53, 54).

Ultra high-performance liquid chromatography total phenolic contents (free+bound) declined with kernel maturity in white and orange grains whereas in red maize the contents first decreased from S1 to S2, and then increased at S3. Concentrations at the physiological maturity stage were higher in the case of the orange and red maize type (223.9 and 229.8 mg/100 g DW, for the orange and red maize, respectively) than results obtained by Fuentes-Cardenas et al. (19) in *Cabanita* race (134.3 and 190.9 mg/100 g DW, for the orange and red maize, respectively). However, above authors reported higher total phenolic contents in the white maize type (206 mg/100 g DW) than in the current research (162.2 mg/100 g DW). Differences in the postharvest treatments and the agroecological conditions for the growth of *Cabanita* maize may explain such variations. Generally, phenolic contents measured with the Folin–Ciocalteu method showed the same trend as those analyzed with the UHPLC method. However, the lack of specificity of the Folin–Ciocalteu method may be associated with differences observed specially in results from the free phenolic fraction (55).

### 3.3. Carotenoid contents and profiles of *Cabanita* maize types at different maturity stages

*Cabanita* maize types at different maturity stages were also evaluated in terms of their carotenoid composition (Table 3, Supplementary Figures S10–S12). In contrast to the variable effect of studied factors (M and S) on phenolic compounds, carotenoid contents were highly influenced by the maize type. Only xanthophylls were found in all *Cabanita* samples while no carotenes were detected. Moreover, different profiles were observed among studied *Cabanita* maize groups. White and red maize had similar profiles and all-trans-neoxanthin, neoxanthin isomer (~13-*cis*-neoxanthin), all-*trans*-zeaxanthin, and a lutein isomer (~13-*cis*-lutein) were the major carotenoids. All-*trans*-lutein and all-*trans*-zeaxanthin were the main compounds in the orange maize, followed by ~13-*cis*-lutein, and neoxanthin compounds. *β*-cryptoxanthin isomers along with some unidentified carotenoids (2, 3) were only detected in this maize type. The concentrations of all mentioned carotenoids in orange maize were higher than values found in white and red types. A violaxanthin isomer (~9-*cis*-violaxanthin) was detected in white and orange maize at all maturity stages, and only at S3 in the red grain. These xanthophylls diversity may be based on the fact that *β*-cryptoxanthin is the metabolic precursor of zeaxanthin which is further metabolized to violaxanthin and then to neoxanthin (56). Several studies have confirmed that predominant carotenoid compounds in maize are generally lutein, zeaxanthin, *β*-cryptoxanthin along with other minor xanthophylls such as zeinoxanthin, antheraxanthin, violaxanthin, neoxanthin, and their isomers (23, 57–59). However, carotene compounds such as α-carotene and β-carotene have been also reported in comparable concentrations in yellow maize varieties (44, 60). Liu et al. (23) reported that two genotypes of Chinese sweet maize showed variable carotenoid profiles and contents during the grain maturation from 10 to 30 DAP. This indicates an important influence of genetic factors and the kernel maturity stage (61).

**Table 3:** Carotenoid profiles and contents (μg/g DW) determined by UHPLC in *Cabanita* maize kernels of different pigmentations and maturity stages.

Compound	White			Red			Orange			*F*-value		
	S1	S2	S3	S1	S2	S3	S1	S2	S3	Maize (M)	Stage (S)	M × S
Neoxanthin isomer^2^ (~13-*cis*-neoxantin)	0.18 ± 0.02c	0.14 ± 0.04c	0.11 ± 0.02c	0.11 ± 0.04d	0.15 ± 0.05c	0.08 ± 0.01c	0.39 ± 0.07a	0.29 ± 0.13b	0.31 ± 0.08ab	38.69^****^	2.42^ns^	1.24^ns^
All-*trans*-neoxanthin^2^	0.19 ± 0.03b	0.15 ± 0.11b	0.22 ± 0.01b	0.16 ± 0.16b	0.17 ± 0.07b	0.18 ± 0.02b	0.17 ± 0.11b	0.22 ± 0.11b	0.37 ± 0.12a	2.79^ns^	2.76^ns^	1.21^ns^
Unidentified carotenoid-1^2^	0.05 ± 0.02	ND^1^	ND	ND	ND	ND	ND	ND	ND			
Violaxanthin isomer^2^ (~9-*cis*-violaxanthin)	0.06 ± 0.02b	0.04 ± 0.02b	0.05 ± 0.01b	ND	ND	0.04 ± 0.02b	0.13 ± 0.04a	0.14 ± 0.06a	0.13 ± 0.05ab			
Unidentified carotenoid-2^2^	ND	ND	ND	ND	ND	ND	0.14 ± 0.05b	0.26 ± 0.04a	0.17 ± 0.06b			
Lutein isomer^2^ (~13-cis-lutein)	0.10 ± 0.03c	0.14 ± 0.08c	0.14 ± 0.05c	0.12 ± 0.06c	0.18 ± 0.11c	0.14 ± 0.03c	0.64 ± 0.27a	0.75 ± 0.24a	0.41 ± 0.04b	48.72^****^	2.69^ns^	2.40^ns^
Unidentified carotenoid-3^2^	ND	ND	ND	ND	ND	ND	0.12 ± 0.03a	0.11 ± 0.03a	ND			
Zeaxanthin isomer^3^ (~13-*cis*-zeaxanthin)	ND	ND	ND	ND	0.03 ± 0.01c	ND	0.18 ± 0.05ab	0.21 ± 0.08a	0.12 ± 0.03b			
All-*trans*-lutein	0.14 ± 0.04c	0.07 ± 0.06c	ND	0.06 ± 0.03c	0.03 ± 0.02c	ND	1.48 ± 0.62b	2.00 ± 0.40a	1.02 ± 0.34b			
All-*trans*-zeaxanthin	0.07 ± 0.04c	0.16 ± 0.04c	0.08 ± 0.03c	0.15 ± 0.07c	0.23 ± 0.06bc	0.09 ± 0.03c	1.44 ± 0.35a	1.35 ± 0.20a	0.54 ± 0.22b	155.08^****^	17.18^****^	11.56^****^
Lutein isomer^2^ (~9 or 9′-*cis*-lutein)	ND	ND	ND	ND	ND	ND	ND	ND	0.07 ± 0.03			
*β*-cryptoxanthin isomer^4^ (~13 or 13′-*cis*-*β*-cryptoxanthin)	ND	ND	ND	ND	ND	ND	0.17 ± 0.06a	0.20 ± 0.09a	0.06 ± 0.01b			
*β*-cryptoxanthin isomer^4^ (~9 or 9′-*cis-β*-cryptoxanthin)	ND	ND	ND	ND	ND	ND	0.10 ± 0.04a	0.19 ± 0.12a	ND			
Total carotenoids	0.77 ± 0.10d	0.69 ± 0.22d	0.62 ± 0.10d	0.66 ± 0.28d	0.84 ± 0.27d	0.56 ± 0.11d	4.97 ± 1.26b	5.87 ± 0.73a	3.19 ± 0.61c	209.22 ^****^	10.53^***^	7.21^***^

Different letters in the same row indicate significant statistical differences (***p < 0.001; and ****p < 0.0001); ns, no significant. *F*-values were calculated only in complete dataset per variable. S1, S2, S3 indicate maturity stages. ^1^Non-detected. ^2^Expressed as lutein. ^3^Expressed as zeaxanthin. ^4^Expressed as *β*-cryptoxanthin.

The maturity stage and the interaction of both factors (M × S) significantly influenced the all-*trans*-zeaxanthin, and the total carotenoid contents. Neoxanthin isomer, all-*trans*-neoxanthin, violaxanthin isomer, and ~13-*cis*-lutein did not show significant changes with kernel development in white and red maize types, but all-*trans*-lutein was not detected at S3. Overall, both maize types showed similar total carotenoid concentrations which were somewhat stable along the kernel growth (ranges of 0.77–0.62 μg/g DW and 0.84–0.56 μg/g DW, for the white and red maize, respectively). In the orange *Cabanita*, all-*trans*-lutein increased by ~35% from S1 to S2 (1.48 and 2.0 μg/g DW at S1 and S2, respectively), but then decreased by 50% at S3 (1.02 μg/g DW). All-trans-zeaxanthin remained almost constant from S1 to S2 (1.44 and 1.35 μg/g DW, respectively). However, it declined by 60% at S3 (0.54 μg/g DW). Similar carotenoid reductions at S3 were observed in case of the 13-cis-lutein, 13-*cis*-zeaxanthin, *β*-cryptoxanthin isomers, and the other unidentified compounds. All-trans-neoxanthin and its isomers showed a certain increase at S3 which indicates the downstream metabolic conversion of *β*-cryptoxanthin, and zeaxanthin (56). The contents of lutein, zeaxanthin, α-cryptoxanthin, and *β*-cryptoxanthin have shown to steadily increase with kernel development from 10 to 30 DAP in sweet corn (23). The increase of zeaxanthin and lutein with kernel maturation from 16 to 24 DAP has been also reported in other sweet maize hybrids (62). Variable zeaxanthin and lutein patterns were observed in some zeaxanthin-biofortified sweet maize depending on the genotype and the kernel position on the cob (61). However, an overall lutein and zeaxanthin accumulation was reported in same study (61). In the current research, higher maturity stages were evaluated (from 28–32 to 75–77 DAP) which likely explains contrasting results compared with previous studies. Xu et al. (24) evaluated a yellow maize variety during maturation from 74 to 116 DAS and found that the contents of zeaxanthin decreased at the end of kernel maturity, whereas lutein increased from 74 to 98 DAS to finally decrease at 116 DAS.

The orange maize exhibited higher total carotenoid contents (3.19–5.87 μg/g DW) than white and red maize (0.77–0.62 μg/g DW and 0.84–0.56 μg/g DW, for the white and red maize, respectively). Nevertheless, carotenoids significantly decreased by ~50% at S3 (from 5.87 to 3.19, at S2 and S3, respectively). Fuentes-Cardenas et al. (19) found lower total carotenoid values in the orange *Cabanita* maize type at physiological maturity (1.95 μg/g DW, COM code) than in the present study. In addition, no carotenoids were detected in the corresponding red and white parental seeds (CCR, CAW) (19). Carotenoids are highly sensitive to light, heat, and oxygen, therefore postharvest practices applied by Andean farmers such as the sun-drying of *Cabanita* ears first in the plant and later on the field for undetermined time may lead to the degradation of carotenoid compounds.

Higher total carotenoid amounts than those from current research have been reported mostly in yellow and sweet maize varieties. Xu et al. (24) found concentrations of 22.78–28.76 μg/g DW at different maturity stages in yellow maize. Ranges of 0.55–43.23 μg/g DW and 11.4–24.0 μg/g DW have been shown in sweet maize harvested at maturity stages from 10 to 32 DAP (23, 63). Floury maize types generally show lower carotenoid contents than hard maize classes such as pop, dent, or flint (64). Therefore, it is expected to find lower carotenoid levels in the amylaceous floury *Cabanita* maize (19).

### 3.4. Fatty acid composition of *Cabanita* maize types at different maturity stages

The fatty acid composition of *Cabanita* maize is shown in Table 4. No significant effect was found by the maize type indicating similar fatty acid profiles and contents among all samples. Polyunsaturated fatty acids (PUFA) including linoleic and α-linolenic acids represented the major fatty acid fraction in all *Cabanita* maize types (55–59% of the total fatty acid content). The monounsaturated oleic acid contributed with 21–28% of the total fatty acids, followed by saturated acids (palmitic and stearic acids, 18–21%). Among all detected fatty acids, linoleic and oleic acids were the most abundant compounds in maize kernels (50–54%, and 21–28% for linoleic and oleic acids, respectively).

**Table 4:** Contents and profiles of fatty acids (mg/g DW) in *Cabanita* maize kernels of different pigmentations and maturity stages.

Maize type	Stage	Saturated fatty acids		Unsaturated fatty acids			Total fatty acids
		Palmitic acid	Estearic acid	Oleic acid	Linoleic acid	α-Linolenic acid	
White	S1	5.1 ± 0.9ab	0.9 ± 0.1bcd	5.9 ± 1.3d	14.7 ± 3.2c	1.6 ± 0.1a	28.0 ± 5.3c
	S2	5.1 ± 0.6ab	0.8 ± 0.1 cd	6.6 ± 1.4 cd	15.6 ± 2.7bc	1.3 ± 0.1de	29.3 ± 4.7bc
	S3	5.9 ± 0.9ab	1.1 ± 0.2a	11.0 ± 2.2a	20.5 ± 4.1a	1.3 ± 0.1e	39.8 ± 7.2a
Red	S1	5.3 ± 0.8ab	0.8 ± 0.1 cd	6.6 ± 1.9 cd	15.3 ± 3.3c	1.5 ± 0.1b	29.5 ± 6.1bc
	S2	5.2 ± 0.6ab	0.8 ± 0.1 cd	7.5 ± 0.9bcd	15.2 ± 2.3c	1.4 ± 0.1 cde	30.1 ± 3.8bc
	S3	6.3 ± 1.3a	1.0 ± 0.2ab	9.8 ± 2.5ab	21.1 ± 4.4a	1.30 ± 0.03e	39.5 ± 8.2a
Orange	S1	4.9 ± 0.6b	0.7 ± 0.1d	5.7 ± 0.9d	14.5 ± 1.5c	1.5 ± 0.1b	27.2 ± 2.8c
	S2	6.0 ± 1.0ab	0.9 ± 0.2bc	9.0 ± 1.9abc	20.4 ± 4.5ab	1.4 ± 0.1bcd	37.6 ± 7.6ab
	S3	5.9 ± 0.6ab	0.9 ± 0.1abc	9.6 ± 1.6ab	21.2 ± 2.9a	1.4 ± 0.1bc	39.0 ± 4.8a
*F*-value	Maize (M)	0.35^ns^	0.62^ns^	0.06^ns^	0.97^ns^	2.84^ns^	0.48^ns^
	Stage (S)	3.91^*^	11.72^***^	17.63^****^	10.29^****^	20.45^****^	11.19^***^
	M × S	0.81^ns^	2.01^ns^	1.55^ns^	1.08^ns^	4.24^**^	1.08^ns^

Different letters in the same column indicate significant statistical differences (*p < 0.05; **p < 0.01; ***p < 0.001; and ****p < 0.0001); ns: no significant differences. S1, S2, S3 indicate maturity stages.

Comparable percentages of linoleic acid have been also reported in Mexican subtropical maize populations (41–51%), sweet maize from the United States (50–63%), and maize varieties from Turkey (50–53%) (65–67). In the case of other Peruvian germplasm, similar fatty acids profiles and concentrations were observed in mature native varieties such as *Chullpi*, *Piscorunto*, Giant *Cuzco*, *Sacsa*, and purple, with ranges of 18.3–25.2 mg/g DW and 9.8–14.6 mg/g DW for linoleic and oleic acids, respectively (68). However, α-linolenic acid concentrations were almost 2 to 3.5-fold higher in *Cabanita* samples (1.3–1.4 mg/g DW, at S3 maturity stage) than in the other Peruvian varieties (0.4–0.7 mg/g DW) (68). Lower α-linolenic acid percentages have been also reported in sweet maize (1.7–2.1%) compared with *Cabanita* maize (3.2–3.7%) (66). Furthermore, this fatty acid was not even detected in several Korean maize hybrids (69). The increase of α-linolenic acid from 0.61 to 4.93% along with the oil contents have been obtained after a long-term breeding process of Mexican maize (65). The contribution of this fatty acid in relation to the total fatty acid content was higher at early maturity stages in *Cabanita* samples (5.1–5.8%).

Linoleic (ω-6) and α-linolenic acids (ω-3) are essential fatty acids that cannot be synthesized by humans (70). After the ingestion, α-linolenic acid is transformed into long-chain ω-3 PUFAs such as docosahexaenoic and eicosapentaenoic acids which play important roles within the organism (71). Dietary α-linolenic acid, and its ω-3 metabolic derivatives have been reported to show antioxidant and anti-inflammatory properties with potential for the prevention of brain malfunction, and cardiovascular disease (72–74). Moreover, diets with ω-6:ω-3 ratios close to 1:1 have been associated with less incidence of chronic diseases including diabetes and cardiovascular diseases (70). Higher ω-6:ω-3 ratios have been found in unbred maize cultivars from Mexico (59–80:1), sweet maize from US (29–33:1), and Peruvian germplasm from other races (36–50:1) in comparison with ratios found in evaluated *Cabanita* maize at physiological maturity stage (15–16:1) (65, 66, 68). Genetic factors may play a role on observed differences. In addition, differences in the agroecological and post-harvest management conditions may also be involved. Based on the current results, *Cabanita* maize shows potential as a dietary source of health relevant PUFAs.

The maturity stage had a strong influence on fatty acid variability in all *Cabanita* types (Table 4). Saturated acids slightly increased at S3, but their proportions with respect to the total fatty acid contents decreased in all cases from 18 to 15% on average. Contents and percentages of stearic acid almost remained constant with maturation. Oleic acid increased from 21 to 22% at S1 to 25–28% at S3. Linoleic acid concentrations also increased and were high at S3, but their percentages in relation to the total fatty acid contents showed almost no variation with kernel growth (from 52 to 51%, from 52 to 53%, and from 53 to 54% for white, red and orange maize, respectively). The α-linolenic acid concentrations and percentages decreased from S1 to S3 (5.1–5.8% to 3.2–3.7%). Palmitoyl-CoA is a metabolic precursor of palmitic acid, and of stearoyl-CoA which in turn serves as a precursor of oleic, linoleic, and linolenic acids biosynthesis *via* several desaturase enzymes (56). A possible metabolic change toward the biosynthesis of oleic and linoleic acids instead of palmitic acid may explain its percentage decrease with maturity. It is noteworthy that ω-6:ω-3 ratios are lower at S1 stage (9.2–10.4:1) than at S3 (15–16:1) indicating better PUFAs balance when *Cabanita* maize is at milk stage.

### 3.5. *In vitro* health-relevant functionality of *Cabanita* maize types at different maturity stages

#### 3.5.1. DPPH and ABTS antioxidant capacity

The antioxidant capacity was evaluated with two different *in vitro* methods and only in the bioavailable-relevant soluble hydrophilic and lipophilic fractions of *Cabanita* maize samples (Table 5). The interaction of maize type (M) and the maturity stage (S) was not significant in all cases; however, all variables were influenced by S. The DPPH hydrophilic antioxidant capacity (DPPH-HF) declined with grain growth and there were differences depending on the maize type (M significant). Orange *Cabanita* showed higher values (422.3–821.7 μmol TE/100 g DW) than white and red types (310.7–490.6 and 308.9–520.2 μmol TE/100 g DW, for the white and red maize, respectively). The DPPH-HF decreased from S1 to S3 by 37, 41 and 49% in the white, red, and orange maize, respectively. The opposite trend was observed in case of the DPPH lipophilic antioxidant capacity (DPPH-LF). Values increased from S1 to S3 around 3.4 and 4.8-fold in the orange and red maize, respectively. In the white maize, the DPPH-LF was not detected at S1, but then it increased to 7.8 and 20.8 μmol TE/100 g DW with kernel development at S2 and S3, respectively.

**Table 5:** *In vitro* antioxidant capacity (μmol TE/100 g DW) in *Cabanita* maize kernels of different pigmentations and maturity stages.

Maize type	Stage	Inhibition of DPPH		Inhibition of ABTS	
		HF	LF	HF	LF
White	S1	490.6 ± 57.3bcd	ND	2065.2 ± 98.7ab	36.4 ± 5.0f
	S2	313.5 ± 79.2 cd	7.8 ± 4.6 cd	1959.7 ± 278.1ab	45.1 ± 7.0ef
	S3	310.7 ± 20.7d	20.8 ± 3.5ab	1009.0 ± 86.0d	70.0 ± 3.7bc
Red	S1	520.2 ± 329.6bc	4.1 ± 3.6d	2012.3 ± 121.8ab	40.6 ± 3.0ef
	S2	507.5 ± 106.0bcd	14.3 ± 7.7bc	1819.8 ± 138.6b	48.4 ± 12.9de
	S3	308.9 ± 150.8d	19.9 ± 12.5ab	1297.5 ± 353.1c	81.0 ± 15.0b
Orange	S1	821.7 ± 114.5a	8.0 ± 3.4 cd	2194.4 ± 121.5a	58.9 ± 5.5 cd
	S2	582.2 ± 135.8b	12.7 ± 4.5bcd	1949.6 ± 185.2ab	71.7 ± 1.6b
	S3	422.3 ± 53.1bcd	27.5 ± 4.1a	1085.2 ± 45.9 cd	94.2 ± 6.4a
*F*-value	Maize (M)	8.51^**^		0.38^ns^	30.91^****^
	Stage (S)	10.05^***^		92.36^****^	68.17^****^
	M × S	1.24^ns^		2.01^ns^	0.50^ns^

Different letters in the same column indicate significant statistical differences (*p < 0.05; **p < 0.01; ***p < 0.001; and ****p < 0.0001); ns, no significant. *F*-values were calculated only in complete dataset per variable. HF, hydrophilic fraction; LF, lipophilic fraction. S1, S2, S3 indicate maturity stages.

The ABTS hydrophilic antioxidant capacity (ABTS-HF) also decreased with kernel maturity in a range of 36–51%, similarly as in the case of the DPPH-HF. However, the maize type did not show an important effect. Ranges were almost comparable among all *Cabanita* types at all maturity stages (1009.0–2065.2 μmol TE/100 g DW, 1297.5–2012.3 μmol TE/100 g DW, and 1085.2–2194.4 μmol TE/100 g DW, for the white, red, and orange maize, respectively). In addition, the ABTS lipophilic antioxidant capacity (ABTS-LF) increased 1.6–2.0-fold from S1 to S3 as also was noticed in case of the DPPH-LF. The orange maize exhibited the highest value at S3 (94.2 μmol TE/100 g DW) among samples (M significant).

The hydrophilic extracts strongly inhibited both free radicals more than lipophilic fractions indicating higher contents of hydrophilic antioxidants such as soluble polyphenols. In fact, higher concentrations of total free phenolic compounds have been determined in this study in comparison with the total carotenoid contents (ranges of 10–65 mg/100 g DW and 0.56–5.87 μg/g DW, for the total free phenolic and total carotenoid contents, respectively). The UHPLC total free phenolic contents highly correlated with the antioxidant capacity (*r* = 0.7709 and *r* = 0.7863, *p* < 0.05 for the DPPH-HF and ABTS-HF, respectively). Hydroxybenzoic acid compounds such as vanillic acid derivatives showed a positive correlation with this property (*r* = 0.5740 and *r* = 0.7502, *p* < 0.05 for the DPPH-HF and ABTS-HF, respectively). Likewise, the HBA-1 compound, and the total HBA contents were correlated with the antioxidant capacity measured with both methods (*r* = 0.5790 and 0.7962, *p* < 0.05 for the DPPH-HF and ABTS-HF, respectively in case of the HBA-1; and *r* = 0.5900 and *r* = 0.7987, *p* < 0.05 for the DPPH-HF and ABTS-HF, respectively in case of the total HBA contents). Moreover, free luteolin derivatives also contributed to the antioxidant capacity in the orange maize group (*r* = 0.6246, *p* < 0.05 for the DPPH-HF). Consistent with the current study, soluble phenolic compounds were correlated with high antioxidant capacity evaluated with the ferric reducing antioxidant power (FRAP) and DPPH methods in several Italian maize landraces (75). Flavonoids including anthocyanins have shown to highly contribute to the free radical antioxidant capacity in Mexican red and purple-pigmented maize (76, 77). In the current study, no correlation was found between the total anthocyanin contents and the antioxidant capacity in *Cabanita* red maize which may be due to its lower anthocyanin ranges compared to HBA concentrations specially at S1 and S2 stages.

In case of the lipophilic antioxidant capacity, a moderate correlation was found between this functional quality and the total carotenoid contents (*r* = 0.5354, *p* < 0.05 with the DPPH method). Other lipophilic compounds such as tocopherols and tocotrienols common in the germ of maize grains but not analyzed in the current study may also play a role (17). Some tocopherol compounds from spelt grain (*Triticum spelta*) have shown significant correlation with the antioxidant capacity measured with the DPPH method (78).

A continuous decrease of the DPPH and FRAP antioxidant capacity was observed in the soluble phenolic fractions from yellow maize at different developmental stages (from 74 to 116 DAS) (24). Hu and Xu (37) and Giordano et al. (39) reported that the free radical inhibitory activity along the kernel growth stages was highly variable depending on the maize variety. An increase of the antioxidant response with kernel maturity were reported in sweet and yellow maize in maturity periods shorter (17–25 DAP and 15–48 DAP, respectively) than in the current research (28–77 DAP) (22, 79). Furthermore, Liu et al. (23) observed that the lipophilic oxygen radical absorbance antioxidant capacity (ORAC) increased with grain maturity (10–30 DAP) in sweet maize showing correlation with the total and individual carotenoids such as lutein and zeaxanthin. Differences in the current results from those of above studies indicates an important influence of genetic factors, the maturity stage, and the origin of maize.

In a previous study with Peruvian white, red, and orange *Cabanita* maize, Fuentes-Cardenas et al. (19) pointed out that hydrophilic compounds strongly contributed to the *in vitro* antioxidant capacity (DPPH and ABTS methods) than lipophilic fractions like this study. Nevertheless, lower ABTS-HF was reported by above authors (566.3–685.4 μmol TE/100 g DW) than in this research (1009.0–1297.5 μmol TE/100 g DW) at physiological maturity stage. This may suggest that postharvest management also plays a role in the observed bioactive variability and associated functional quality.

#### 3.5.2. Inhibitory activity against α-amylase and α-glucosidase enzymes

The intake of natural inhibitors of key intestinal carbohydrate-hydrolyzing enzymes such as α-amylase and α-glucosidase may represent an important dietary strategy for hyperglycemia management relevant for the type-2 diabetes prevention (80–82). Table 6 shows the potential *in vitro* inhibitory activity of the soluble hydrophilic and lipophilic fractions from *Cabanita* maize samples against α-amylase and α-glucosidase enzymes.

**Table 6:** *In vitro* inhibitory activity against α-amylase and α-glucosidase in *Cabanita* maize kernels of different pigmentations and maturity stages.

Maize type	Stage	α-Glucosidase inhibitory activity (%)						α-Amylase inhibitory activity (%)					
		3 mg^1^		6 mg		10 mg		25 mg		62 mg		125 mg	
		HF	LF	HF	LF	HF	LF	HF	LF	HF	LF	HF	LF
White	S1	17.2 ± 3.5a	6.1 ± 4.3a	31.3 ± 4.0a	7.5 ± 2.6ab	40.0 ± 6.3a	8.9 ± 2.0b	7.0 ± 3.0a	ND^2^	18.6 ± 8.3a	ND	55.0 ± 25.4a	4.3 ± 5.9
	S2	10.7 ± 4.2 cd	4.7 ± 4.9a	16.6 ± 4.8de	7.1 ± 1.7ab	20.2 ± 4.9 cd	11.9 ± 3.3ab	1.4 ± 1.6b	ND	4.1 ± 3.0c	ND	13.5 ± 4.4b	ND
	S3	6.5 ± 1.4d	5.6 ± 1.6a	10.9 ± 2.9e	7.3 ± 0.3ab	14.8 ± 2.8d	8.6 ± 0.7b	ND	ND	1.8 ± 2.7c	ND	8.1 ± 1.7b	ND
Red	S1	18.4 ± 3.3a	3.4 ± 2.7a	28.0 ± 5.4ab	8.1 ± 4.2ab	32.3 ± 6.9b	12.2 ± 4.0ab	2.4 ± 3.4b	ND	6.6 ± 5.9bc	ND	18.2 ± 8.9b	ND
	S2	16.1 ± 1.7ab	3.8 ± 2.9a	23.0 ± 4.3bc	8.7 ± 2.5ab	31.8 ± 2.8b	12.3 ± 2.8ab	2.0 ± 2.8b	ND	8.0 ± 3.0bc	ND	16.3 ± 3.2b	ND
	S3	7.5 ± 3.8 cd	5.5 ± 4.6a	15.2 ± 6.8de	9.7 ± 3.0a	20.2 ± 7.6 cd	12.2 ± 3.5ab	2.1 ± 2.6b	ND	4.2 ± 4.5c	ND	8.3 ± 2.9b	ND
Orange	S1	20.5 ± 2.3a	4.9 ± 5.8a	30.0 ± 3.4a	5.7 ± 6.2ab	41.0 ± 2.1a	8.6 ± 5.8b	6.8 ± 1.8a	ND	12.8 ± 4.4ab	ND	19.0 ± 3.2b	ND
	S2	10.8 ± 3.0 cd	1.9 ± 2.9a	17.4 ± 3.5 cd	4.6 ± 3.2b	22.7 ± 4.1c	10.2 ± 2.2ab	ND	ND	3.9 ± 1.6c	ND	9.3 ± 4.3b	ND
	S3	12.1 ± 4.2bc	3.4 ± 2.8a	18.3 ± 1.9 cd	8.7 ± 1.3ab	22.6 ± 4.1c	15.1 ± 5.3a	ND	ND	3.0 ± 2.0c	ND	7.9 ± 0.5b	ND
*F*-value	Maize (M)	3.02^ns^	0.88^ns^	1.22^ns^	1.86^ns^	1.99^ns^	1.39^ns^			0.66^ns^		7.11^**^	
	Stage (S)	30.13^****^	0.48^ns^	38.26^****^	1.08^ns^	43.89****	1.09^ns^			15.76^****^		19.26^****^	
	M × S	2.60^ns^	0.29^ns^	2.48^ns^	0.50^ns^	5.12^**^	1.71^ns^			4.10^*^		6.78^***^	

Different letters in the same column indicate significant statistical differences (*p < 0.05; **p < 0.01; ***p < 0.001; and ****p < 0.0001); ns, no significant. *F*-values were calculated only in complete dataset per variable. HF, hydrophilic fraction; LF, lipophilic fraction. ^1^Sample dose. ^2^Non-detected. S1, S2, S3 indicate maturity stages.

All hydrophilic (HF) and lipophilic (LF) maize extracts inhibited the α-glucosidase enzyme in a sample dose dependent manner (3–10 mg). However, HF extracts showed higher inhibition than LF fractions. The type of maize (M) was not significant on the α-glucosidase inhibitory activity of both HF and LF fractions at all evaluated sample doses. This indicates similar inhibitory potential among all *Cabanita* maize types. No effect of the M x S interaction was found on results from HF and LF fractions, except at 10 mg (HF). In this case, HF fractions from white and orange maize exhibited greater inhibition than red maize at S1 (40.0, 41.0, and 32.3%, for the white, orange, and red maize, respectively). Nonetheless, the inhibitory activity of red maize was higher than white and orange at S2 (31.8, 20.2, 22.7%, for the red, white, and orange samples, respectively), whereas results of all HF fractions were almost similar at S3.

The maturity stage (S) had a significant influence on the HF inhibitory activity at all sample doses. At 10 mg, this property decreased with kernel development (from 28–33 to 75–76 DAP) by 63, 37 and 45% in the white, red, and orange group, respectively. This reduction occurred at all sample doses, indicating that hydrophilic inhibitors may be related to the soluble phenolic fraction which also declined with kernel maturity as previously stated. This *in vitro* functional quality had high correlation with the free UHPLC soluble phenolic compounds at all sample doses (*r* = 0.7386, *r* = 0.8064, and *r* = 0.8545, *p* < 0.05 at 3, 6, and 10 mg sample dose, respectively). Specific phenolic compounds such as the vanillic acid derivatives, HBA-1, and the total HBA contents positively correlated with the inhibitory potential of *Cabanita* maize samples (*r* = 0.7962, *r* = 0.7728, and *r* = 0.7961, *p* < 0.05, respectively, 10 mg sample dose). Among free HCA compounds, ferulic acid derivatives also showed a significant correlation (*r* = 0.6597, *p* < 0.05, 10 mg sample dose). In case of LF fractions, results were not influenced by S, showing comparable α-glucosidase inhibition during the grain growth. However, a certain increase (from 5.7 to 8.7% and from 8.6 to 15.1% at 6, and 10 mg of sample dose, respectively) was observed in the orange maize. No significant correlations were found between the LF α-glucosidase inhibitory activity, and any metabolites measured in the current study.

The α-amylase enzyme, relevant for the hydrolysis of α-1,4-glucan polysaccharides into maltose and maltooligosaccharides (83), was inhibited only by HF maize fractions in a dose-dependent manner (Table 6). All values decreased with kernel maturity (S significant) similarly as in the case of the α-glucosidase inhibitory activity. When the maturity stage changed from S1 to S3, the white maize (125 mg dose) showed the highest loss of the inhibitory potential (around 85%), followed by the red, and orange maize (~54 and 58% in red and orange maize, respectively). Both M × S interaction and M factors greatly influenced the α-amylase inhibition. White maize samples had higher α-amylase inhibition at S1 among *Cabanita* maize types. The α-amylase inhibitory potential positively correlated with the free UHPLC total phenolic contents (*r* = 0.6358 and *r* = 0.6574, *p* < 0.05, at 62 and 125 mg of sample dose, respectively). Furthermore, all HBA compounds and free ferulic acid derivatives were correlated with this *in vitro* functional property (*r* = 0.5278–0.6471 at all sample doses, and *r* = 0.5340–0.5599 at 62–125 mg, respectively).

Different studies have highlighted the role of phenolic compounds for hyperglycemia prevention and countering associated oxidative complications through several mechanisms including the modulation of gastric enzymes at intestinal level (84–87). Cereal-derived phenolic acids including several HBA, and HCA compounds have shown inhibitory potential against the intestinal α-glucosidase enzyme which was highly dependent on the number of hydroxyl and methoxy groups in their structure (88). HBA derivatives such as methyl vanillate (a vanillic acid derivative), syringic acid, and vanillic acid from Thai colored rice showed higher inhibition of α-glucosidase than on α-amylase with a mixed-type inhibition mode against α-glucosidase (89). In same study, *in silico* analysis revealed that the inhibition involved the molecular interaction between HBA and the binding sites of digestive enzymes through 3–4 hydrogen bonds depending on the phenolic compound and the enzyme (89). Conjugated hydroxycinnamic acids amides (HCCA) identified in maize and in other grains from the *Poaceae* family grains such as *N,N*-di-feruloylputrescine, *N-p*-coumaroyl-*N*-feruloylputrescine have also been targeted as α-glucosidase inhibitors (90, 91). Recently, another ferulic acid derivative named 6’-*O*-feruloylsucrose isolated from black rice bran showed high α-glucosidase inhibition when *in vitro* and *in silico* studies were performed (92).

Based on above information, the free phenolic fraction including HBA, and some HCA derivatives likely involving ferulic acid derivatives detected in the current research may explain the *in vitro* anti-hyperglycemia potential of *Cabanita* maize HF extracts. However, other polar compounds may also be involved. Some compounds detected with the untargeted GC–MS analysis as will be discussed in next section, have shown direct correlation with results from both enzymatic assays. Alcohol sugars including d-sorbitol, meso-erythritol and phytosterols such as campesterol, and sitosterol positively correlated with the α-amylase (*r* = 0.7588, *r* = 0.6556, *r* = 0.6235, and *r* = 0.7850, *p* < 0.05, at 125 mg dose respectively) and α-glucosidase inhibitory activities (*r* = 0.7275, *r* = 0.7062, *r* = 0.6965, and *r* = 0.7218, *p* < 0.05, at 10 mg, respectively). Some studies have pointed out the role of erythritol and triterpenoid compounds for the management of postprandial blood glucose through the inhibition of α-glucosidase enzyme (93, 94). Stigmasterol among other phytochemicals identified in maize silk have been indicated as potential inhibitors of both α-glucosidase and α-amylase enzymes (95).

The observed LF α-glucosidase inhibitory activity may be ascribed to lipophilic compounds that increase in maize kernel with maturity time specially in case of the orange maize. The contents of α-tocopherol, *β*-tocotrienol, *γ*-tocotrienol, and *δ*-tocotrienol had increased with maturity in grains of *Amaranthus cruentus* (96). Lipophilic extracts from *Vicia fava* L. seeds containing *α*-tocopherol and *γ*-tocopherol compounds exhibited high *α*-glucosidase inhibition (97). It is possible that the inhibitory activity of *Cabanita* maize extracts against digestive enzymes may be due to the synergistic action of HL and LF compounds as also was reported by Parizad et al. (98) in several pigmented cereals. Future studies are necessary to reveal the identity of HF and LF compounds from *Cabanita* maize and their molecular mechanisms of inhibition against hyperglycemia-relevant enzymes.

*α*-Amylase inhibitory activity results from this study are comparable with those reported by Ranilla et al. (20) in mature *Cabanita* maize kernels from Peru (8.9–10.2%, at 125 mg sample dose). However, higher *α*-glucosidase inhibitory activities were obtained by above authors (34.9–40.8%, at 12.5 mg of sample dose) which may be linked to the higher sample doses evaluated. On the other hand, lower inhibitory activities against *α*-amylase and *α*-glucosidase were found in the free phenolic fraction from Chinese fresh waxy maize harvested at milk stage (~28–72% and ~32–48% for the *α*-amylase and α-glucosidase inhibitory activities, respectively, at 1,000 mg sample dose) than in current study at similar maturity stage (2.4–55.0% at 25–125 mg sample dose, and 17.2–41.0% at 3–10 mg sample dose for the *α*-amylase and *α*-glucosidase inhibitory activities, respectively) (99). Several studies have shown the anti-hyperglycemic potential of maize from different origins (36, 98, 100). Nevertheless, the impact of the maturity stage on this functional property in selected samples from the Peruvian *Cabanita* maize diversity is shown for the first time in this study.

### 3.6. Primary polar metabolites analysis by GC–MS of *Cabanita* maize types at different maturity stages and principal component analysis

**Figure 4:**
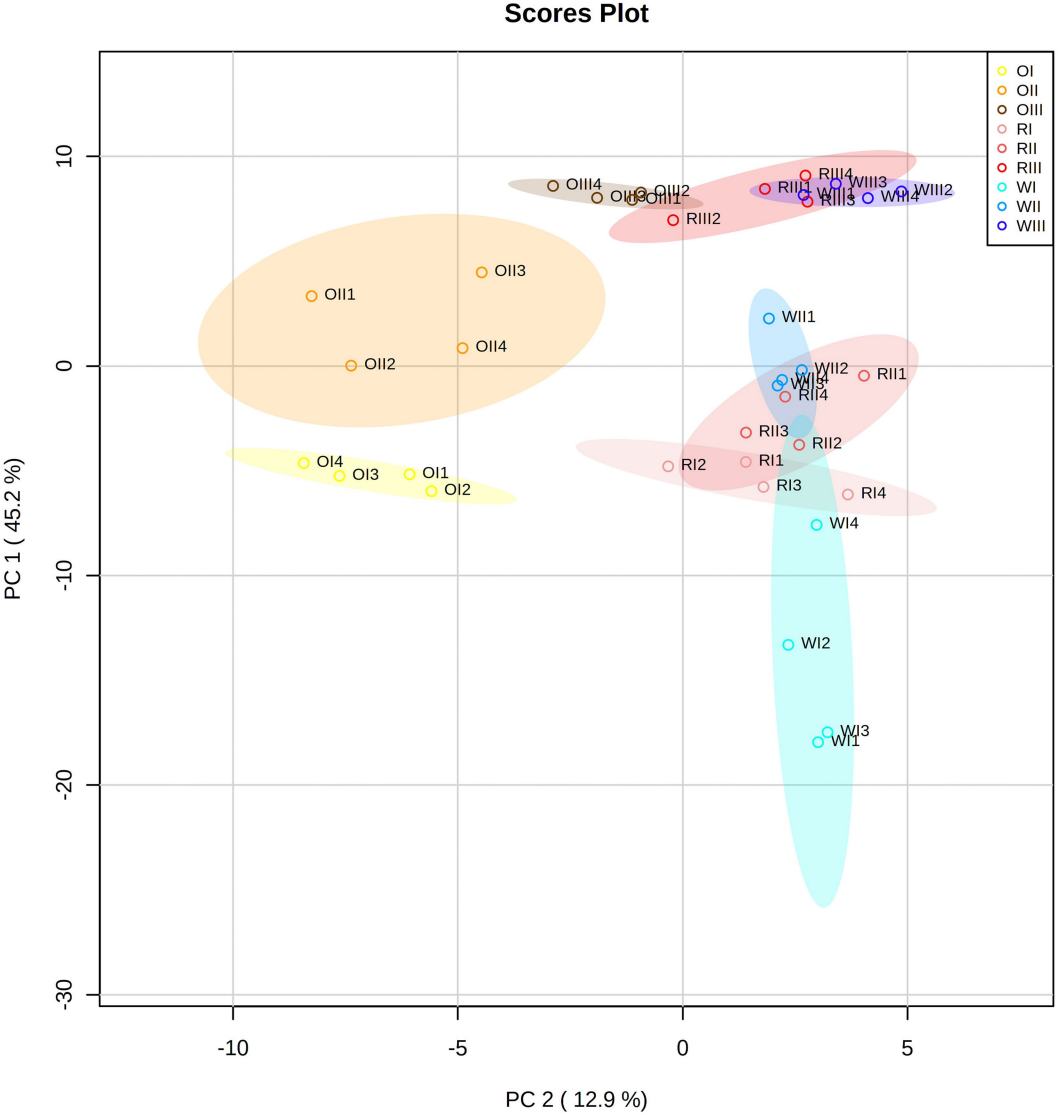
Principal component analysis (PCA) score plot of all data from white (W), red (R), and orange (O) *Cabanita* race at different maturity stages (I: S1, II: S2, III: S3).

**Figure 5:**
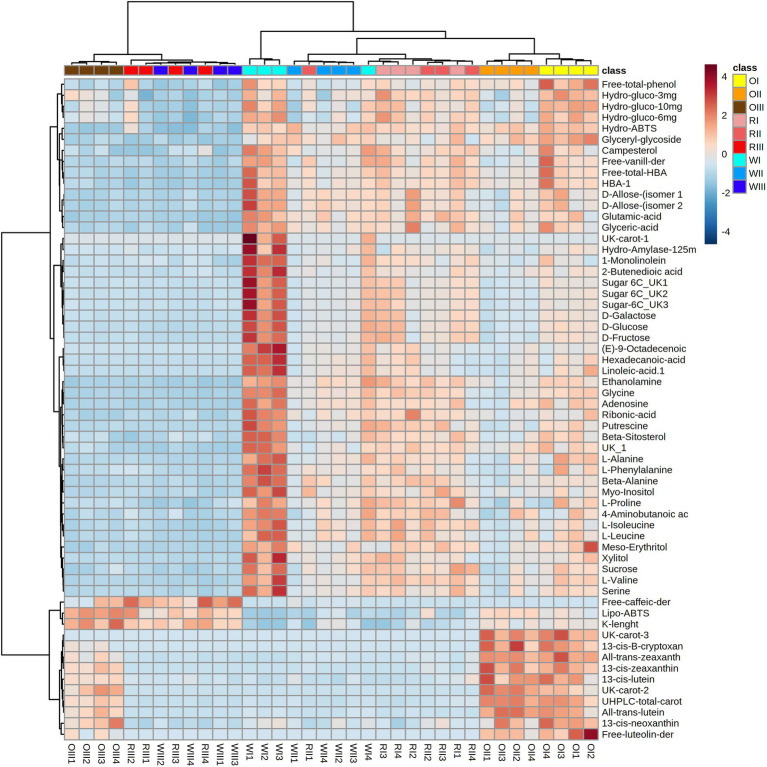
Heat map considering 60 significant variables from white (W), red (R), and orange (O) *Cabanita* race at different maturity stages (I: S1, II: S2, III: S3).

The GC–MS analysis allowed to detect 63 polar metabolites including sugars, amino acids, other nitrogen-containing compounds, free fatty acids, organic acids, sugar alcohols, and phytosterols. A PCA analysis was performed considering all data from the targeted, untargeted metabolomic analyses, and the *in vitro* functional quality to reveal underlying relationships among all variables (Figure 4). The first two principal components from the PCA model explained 58.1% of the total dataset variability. Different groups were observed based on the maize type and maturity stage which were explained by 98 significant variables. The heat map considering the top 60 significant variables is shown in Figure 5. PC 1 (45.2% of explained variance) separated (from top to the bottom) all maize types at S3 (OIII, RIII, and WIII, for the orange, red, and white maize at S3, respectively) from samples at earlier maturity stages (I, II, for S1 and S2, respectively).

Maize samples at S3 were characterized by overall lower concentrations of secondary metabolites (phenolic and carotenoid compounds), along with reduced *in vitro* antioxidant and anti-hyperglycemia potential than maize at S1 and S2. In relation to the primary polar metabolites detected by GC–MS, free monosaccharides (glucose, fructose, galactose), disaccharides (sucrose) among other unidentified sugar molecules decreased with kernel maturity in all maize types (Figure 5). Furthermore, several amino acids (glutamic acid, glycine, alanine, phenylalanine, proline, isoleucine, leucine, valine, and serine) also declined with grain maturity. Simple sugars may have been used as carbon sources for cellular energy metabolism, and the biosynthesis of starch which is known to accumulate in mature cereal grains (101). The decrease of sugar contents with the concurrent increase of starch during grain development was observed by Xu et al. (24) and Saikaew et al. (101) in yellow and purple waxy maize, respectively. Amino acids may have been transformed into proteins or used as metabolic precursors for the synthesis of secondary metabolites (102). The increase of protein with kernel maturation has been reported in maize and rice kernels (101, 103). Among data from all maize types at S3, the orange maize clearly separated and stood out from the red and white groups, whereas some replicates from these last maize types overlapped. This difference was mainly correlated with the highest carotenoid contents in the orange group than in red and white maize.

PC 2 (12.9% of explained data variability) separated orange maize (at all maturity stages) from white and red types (from left to right). The orange maize showed unique flavonoids such as luteolin derivatives and had the highest carotenoid concentrations (especially at S1 and S2) as previously stated. Interestingly, the white maize was different from the other maize types specifically at the S1 stage (Figure 5). Primary metabolites including simple sugars (monosaccharides and sucrose), amino acids, free fatty acids (linoleic and palmitic acids), organic acids (4-aminobutanoic acid or GABA, fumaric acid), amines (putrescine, ethanolamine), myo-inositol and xylitol were more abundant in the white maize at milk stage than in the other maize groups at same maturity period.

Phytosterols such as campesterol and β-sitosterol were detected in all maize groups. The orange and red maize showed comparable campesterol contents, but the white maize exhibited the highest sitosterol abundance at S1. Phytosterols are bioactive compounds with potential for the prevention of cardiovascular diseases because of their cholesterol-lowering properties (104). Both detected phytosterols decreased with grain maturity in all *Cabanita* types. The reduction of *β*-sitosterol has been also observed during *Camellia chekiangoleosa Hu*. seeds development (105). *β*-sitosterol is the key precursor of sitosterol-β-glucoside which play a role on the biosynthesis of cellulose (106). The increase of dietary fibre, which is composed of cellulose, hemicellulose, lignin, among other polymers has been reported in *Amaranthus cruentus* with grain maturity (96). Furthermore, the cell wall feruloylation along with the lignin-cross links of the wheat grain outer layers increased during kernel development (107). This likely explains the decrease of free ferulic acid derivatives and the increase of some cell wall phenolics (bound ferulic acid derivatives) specially in the red and orange maize at S3 maturity stage.

Other detected primary metabolites such as free fatty acids (palmitic acid, linoleic acid), organic acids (glyceric acid, fumaric acid, ribonic acid, GABA), amines (ethanolamine, putrescine), myo-inositol, and alcohol sugars (erythritol, xylitol) were also reduced with grain maturity in all maize types. Free fatty acids may have been metabolized for the synthesis of triacylglycerols (TAG) since the total fatty acid contents (derived from the triacylglycerol saponification) increased with grain maturity in all *Cabanita* groups (Table 4). The increase of the total lipid contents during the grain development of yellow maize has been related to the late embryo formation (24). The reduction of ethanolamine and glyceric acid may have been targeted as precursors of glycerophospholids (components of the cell membranes) during grain growth. Some types of phosphatidilethanolamines esterified with variable fatty acids have increased during wheat kernel filling (108). The reduction of intermediate metabolites involved in the tricarboxylic acid cycle (TCA) such as fumaric acid and GABA-derived succinic acid might reflect a high mitochondrial activity for energy generation during the maize grain development (109). Polyamines including putrescine have essential roles in many biochemical and physiological processes, in particular stress and senescence responses during plant growth and development (110). Putrescine content also decreased with kernel maturity in *Cabanita* maize as observed in other polar metabolites. This polyamine may have been used for the biosynthesis of some HCAAs such as *N-p*-coumaroyl-*N*-feruloylputrescine and caffeoylputrescine which have been detected in mature maize (44). In the current study, *p*-coumaric and caffeic acid derivatives (possibly HCAAs) have increased with *Cabanita* kernel maturation.

## 4. Conclusion

Maize with different kernel pigmentations (white, red, and orange) and representing the diversity of the Peruvian Andean maize race *Cabanita* showed variable primary (polar metabolites), and secondary metabolite composition (phenolic and carotenoid compounds) and were greatly influenced by the grain maturity stage. All maize types showed free HBA and HCA phenolic compounds, but luteolin derivatives and anthocyanins were only detected in orange and red maize, respectively. Major bound phenolic compounds were ferulic acid, followed by ferulic acid derivatives, and *p*-coumaric acid in all *Cabanita* groups. However, orange, and red types had higher bound ferulic acid, and total phenolic contents (free + bound) (223.9–274.4 mg/100 g DW, 193.4–229.8 mg/100 g DW for the orange and red maize, respectively) than the white maize (162.2–225.0 mg/100 g DW). Xanthophylls including lutein, zeaxanthin, neoxanthin, lutein isomer (~13-*cis*-lutein) were detected in all maize types. The orange maize had the highest total carotenoid contents (3.19–5.87 μg/g DW) and contained specific carotenoids such as *β*-cryptoxanthin and zeaxanthin isomers. Most phenolic and carotenoid compounds decreased with kernel maturity in all *Cabanita* maize types. With respect to the primary metabolites, all maize types showed similar fatty acid contents (linoleic acid > oleic acid > palmitic acid > *α*-linolenic acid > stearic acid) which increased with kernel development. Other primary metabolites such as simple sugars, alcohols, amino acids, free fatty acids, organic acids, amines, and phytosterols declined with grain maturity and were overall more abundant in white maize at S1. The *in vitro* antioxidant potential and the inhibitory activity against digestive enzymes (*α*-amylase and *α*-glucosidase) were high in the hydrophilic fractions and correlated with the free phenolic fraction. In general, all *Cabanita* maize types had similar *in vitro* health-relevant functionality which significantly decreased with grain development. Based on above results, recommended harvesting periods for the consumption of the orange and white *Cabanita* would be at S1 and S2 stages due to their higher phenolic, carotenoid contents (in case of the orange type), *in vitro* functional qualities, phytosterol concentrations, and better ω-6:ω-3 PUFAs balance. The red *Cabanita* maize would be more valuable at S3 because of its higher total anthocyanin, and phenolic contents. Nevertheless, the potential changes on *Cabanita* technological and processing characteristics at lower maturity stages should be further evaluated. Current study provides relevant metabolomic and biochemical information that contribute to the characterization of the Andean *Cabanita* maize diversity. Insights from this research would be important for promoting its consumption beyond its mature form as it is currently applied, and for future improvements at postharvest level. Studies at transcriptomic level would help to reveal the mechanisms involved in the metabolic changes related to the secondary and primary metabolites during *Cabanita* maize maturation. Next level studies should focus on improving the nutraceutical and nutritional properties of *Cabanita* maize with its sustainability and consumer-relevant yield characteristics within the context of Andean food systems.

## Data availability statement

The raw data supporting the conclusions of this article will be made available by the authors, without undue reservation.

## Author contributions

LR and GZ conceived and designed the study. LR directed the research and wrote the manuscript. AA-C performed the experiments. RC helped with the experimental work. HH contributed with the direction of crop management and pollination control. MV-V helped with the statistical analysis. HB-G coordinated and helped with the original sample collection. RP developed the untargeted metabolomic and free fatty acid analysis. GZ, RC, RP, HH, and KS critically reviewed the manuscript. All authors contributed to the article and approved the submitted version.

## Funding

This research was supported by PROCIENCIA-CONCYTEC (Peru) under the Basic Research Program E041-2018-01, Contract N°114-2018-FONDECYT.

## Conflict of interest

The authors declare that the research was conducted in the absence of any commercial or financial relationships that could be construed as a potential conflict of interest.

## Publisher’s note

All claims expressed in this article are solely those of the authors and do not necessarily represent those of their affiliated organizations, or those of the publisher, the editors and the reviewers. Any product that may be evaluated in this article, or claim that may be made by its manufacturer, is not guaranteed or endorsed by the publisher.

## Footnotes

^1^ https://www.infostat.com.ar/

^2^ https://www.metaboanalyst.ca/MetaboAnalyst/home.xhtml
