# Supplementary material for: Integrated metabolite analysis and health-relevant in vitro functionality of white, red, and orange maize (Zea mays L.) from the Peruvian Andean race Cabanita at different maturity stages
Source: Front Nutr. 2023 Feb 28;10:1132228. doi: 10.3389/fnut.2023.1132228 (PMC10011086; doi:10.3389/fnut.2023.1132228)
Supplement: Supplementary file 1 [file Data_Sheet_1.docx]

Supplementary Material

1. Supplementary Tables

**Supplementary Table 1.** Physico-chemical characteristics of soil used for the cultivation of *Cabanita* maize

| Characteristic^1^ | Value |
| --- | --- |
| Sand (%) | 55.1 |
| Lime (%) | 27.1 |
| Clay (%) | 17.8 |
| Porosity (%) | 38.0 |
| Water (%) | 7.9 |
| Organic matter (%) | 8.1 |
| pH | 8.0 |
| Electric conductivity (dS/m) | 2.6 |
| Total nitrogen (%) | 0.3 |
| P (ppm) | 166.0 |
| K (ppm) | 3100.0 |
| CO_3_Ca | 2.9 |

^1^ Soil composite sample (5 kg).

**Supplementary Table 2.** Meteorological conditions per month-year during *Cabanita* maize plants growth^1^

| N° | Month | Max Temp (°C) | Min Temp. (°C) | Rainfall  (mm/day) | Relative Humidity (%) | UV Radiation^2^ |
| --- | --- | --- | --- | --- | --- | --- |
| 0 | Nov-2020 | 25.0 | 9.0 | 0 | 42.1 | 8.5 |
| 1 | Dec-2020 | 24.3 | 11.0 | -30 | 55.4 | 8.6 |
| 2 | Jan-2021 | 24.1 | 12.0 | 0.5 | 62.0 | 7.3 |
| 3 | Feb-2021 | 25.2 | 11.6 | -40.3 | 62.9 | 8.3 |
| 4 | Mar-2021 | 24.2 | 11.4 | -29.1 | 68.0 | 6.3 |
| 5 | Apr-2021 | 24.5 | 9.9 | -74.0 | 58.9 | 6.0 |
| 6 | May-2021 | 24.7 | 8.6 | -45.4 | 48.3 | 7.5 |
| 7 | Jun-2021 | 23.8 | 7.2 | 0 | 45.2 | 8.3 |

^1^Values are the average per month. Adapted from data of *La Pampilla* meteorological station (S: 16° 24' 49.66''; W: 71° 32' 4.31''; district of Arequipa, Arequipa province, Peru). National Meteorology and Hydrology Service of Peru (SENAMHI). <https://www.senamhi.gob.pe/main.php?dp=arequipa&p=estaciones>). This was the closest station to the site of the experiment. ^2^Values measured weekly between 11:00 am to 12:00 am.

# Supplementary Figures

#
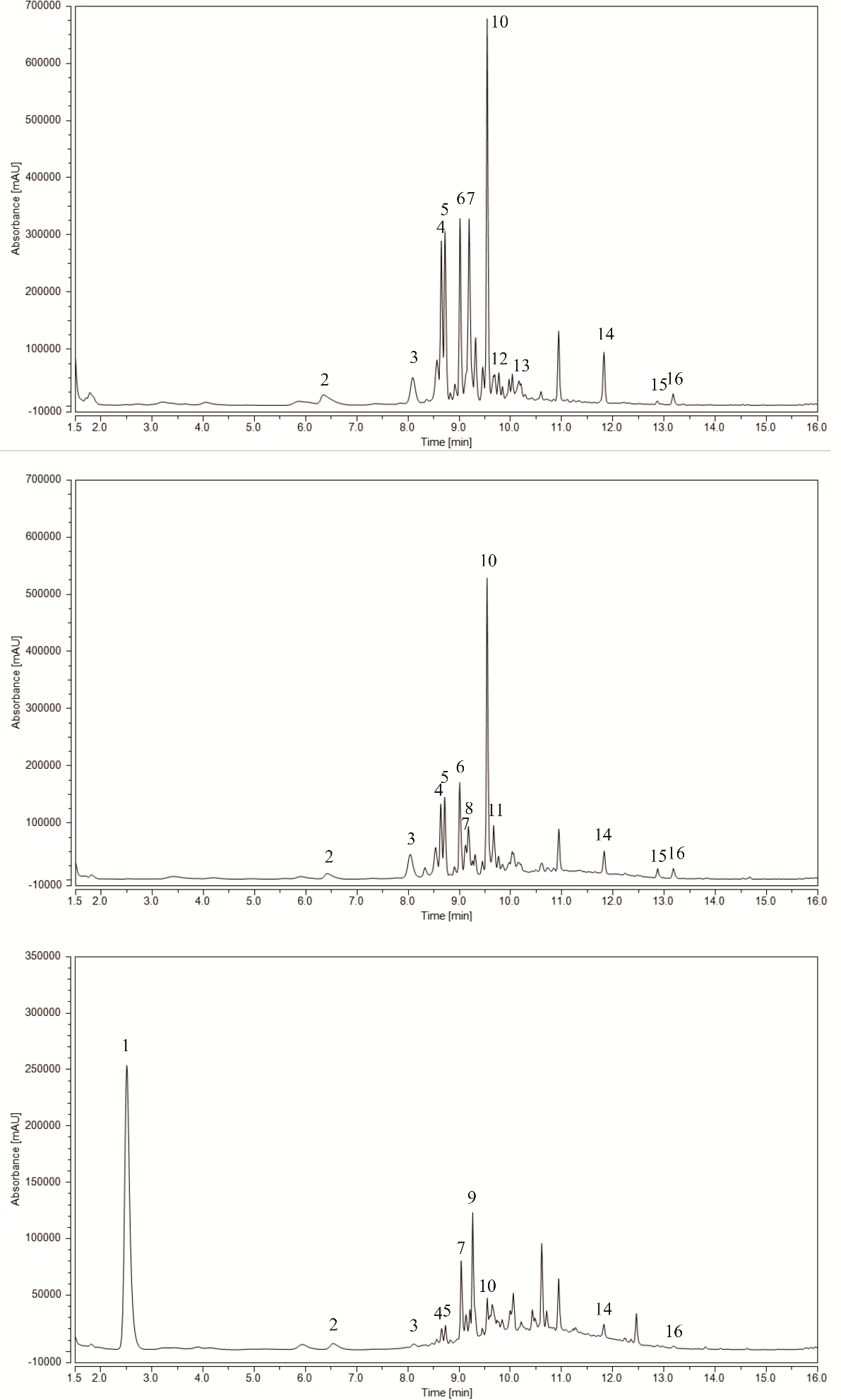


S3

S2

S1

**Supplementary Figure 1.** Chromatograms of hydroxybenzoic acids (HBA) detected at 280 nm (free phenolic fraction) by UHPLC-DAD in white *Cabanita* maize at three maturity stages (S1, S2, S3).


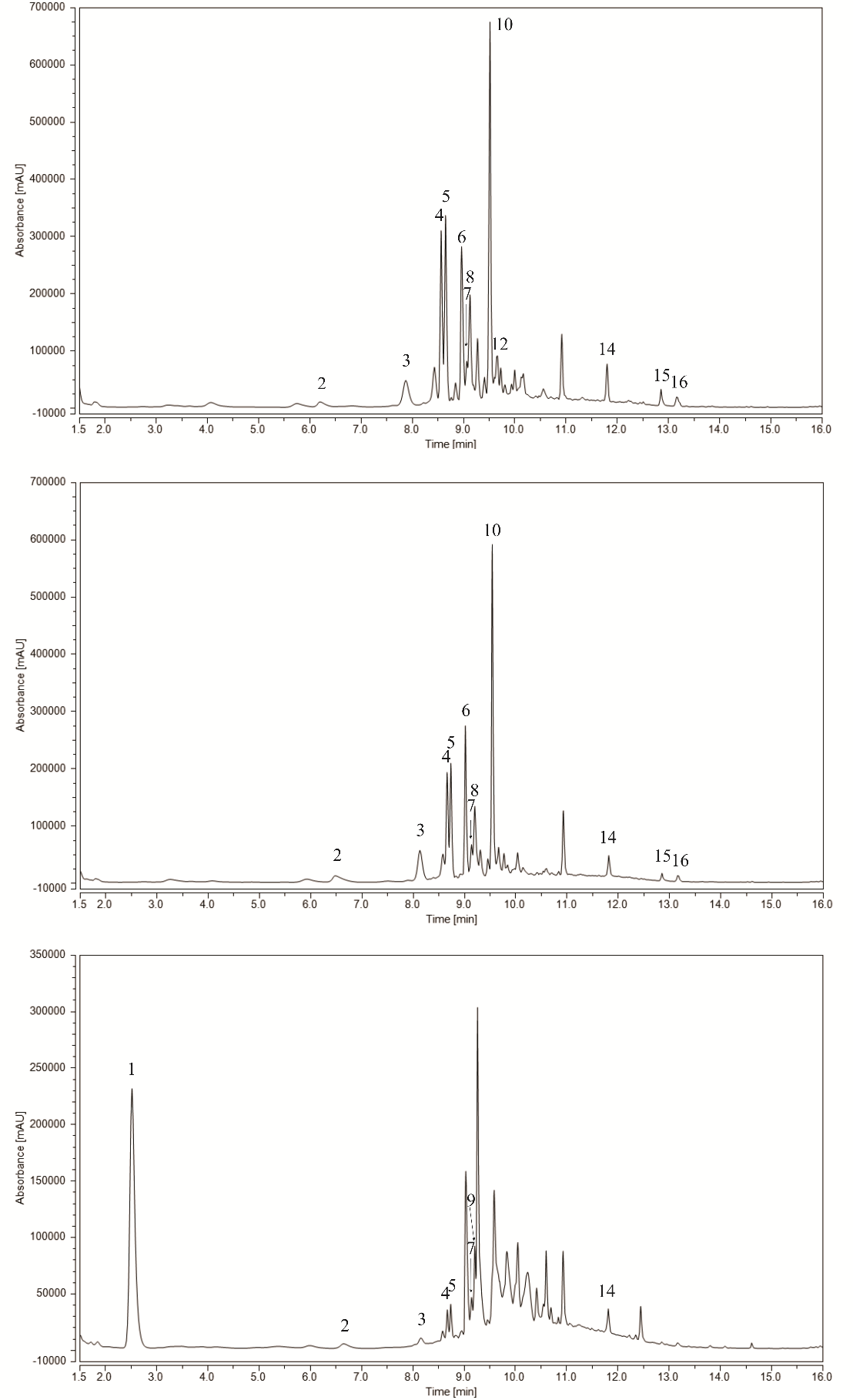


S3

S2

S1

**Supplementary Figure 2.** Chromatograms of hydroxybenzoic acids (HBA) detected at 280 nm (free phenolic fraction) by UHPLC-DAD in red *Cabanita* maize at three maturity stages (S1, S2, S3).


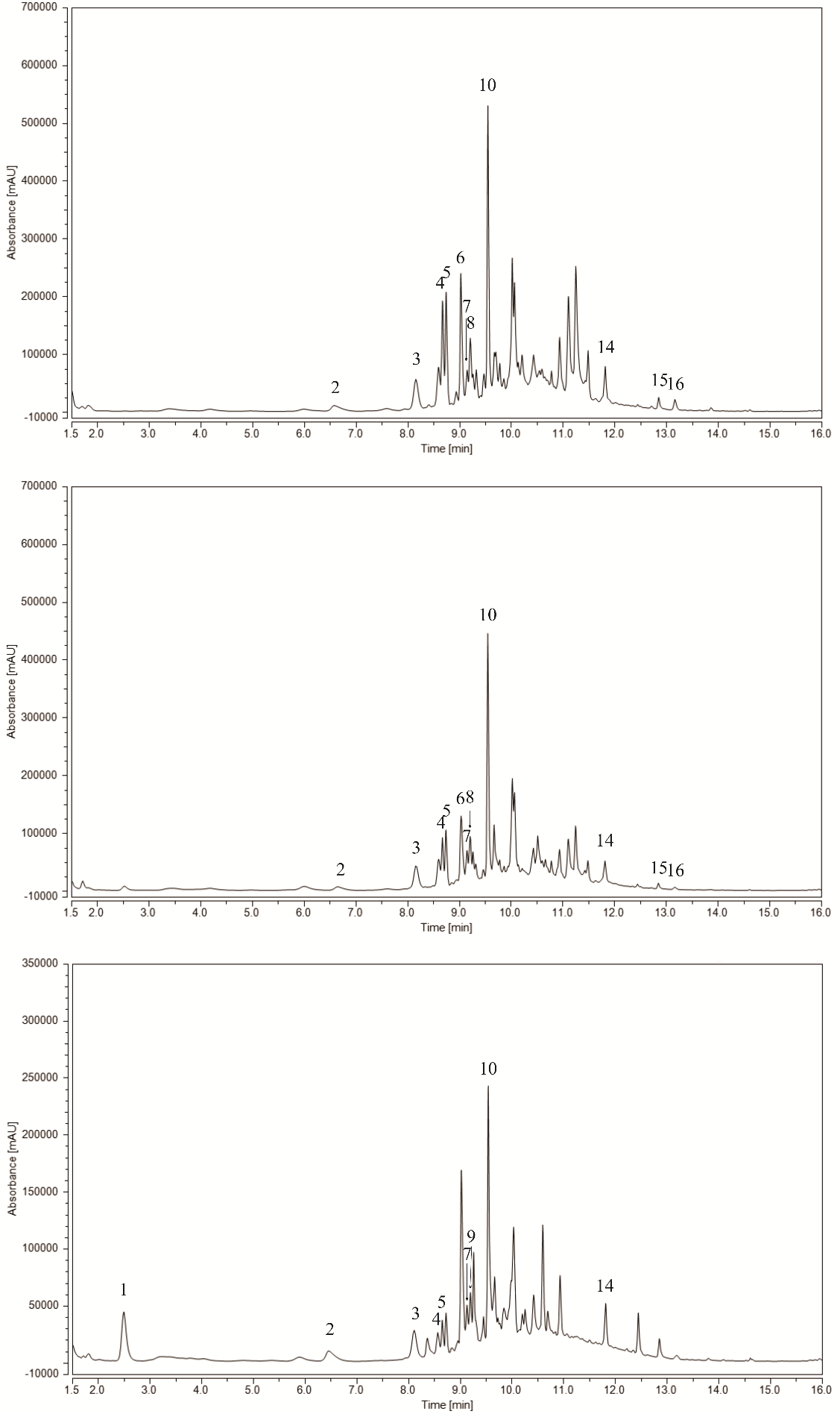


S3

S2

S1

**Supplementary Figure 3.** Chromatograms of hydroxybenzoic acids (HBA) detected at 280 nm (free phenolic fraction) by UHPLC-DAD in orange *Cabanita* maize at three maturity stages (S1, S2, S3).

Supplementary Table 3: Retention times, spectra information, and free phenolic compounds (hydroxybenzoic acids, HBA) detected at 280 nm in white, red, and orange Cabanita maize (Supplementary Figures 1-3).

| Nº Peak | Retention time (min) | λ max (nm) | Compound |
| --- | --- | --- | --- |
| **1** | 2.485-2.518 | 229.64, 284.41 | Unidentified amino acid |
| **2** | 6.198-6.643 | 219.22, 278.78 | HBA derivative 1 |
| **3** | 7.85-8.172 | 218.21, 278.79 | HBA derivative 1 |
| **4** | 8.558-8.677 | 214.97, 249.34, 286.72 | Vanillic acid derivative |
| **5** | 8.647-8.747 | 249.32, 286.76 | Vanillic acid derivative |
| **6** | 8.962-9.032 | 219.11, 279.59, 315.76 | HBA derivative 1 |
| **7** | 9.058-9.153 | 248.32, 289.52 | Vanillic acid derivative |
| **8** | 9.123-9.213 | 219.09, 279.69 | HBA derivative 1 |
| **9** | 9.207-9.270 | 205.2, 265.94 | HBA derivative 2 |
| **10** | 9.51-9.558 | 219.54, 278.88 | HBA derivative 1 |
| **11** | 9.667-9.682 | 218.51, 278.85 | HBA derivative 1 |
| **12** | 9.66-9.702 | 209.46, 269.99 | HBA derivative 2 |
| **13** | 10.203-10.217 | 206.44, 265.09 | HBA derivative 2 |
| **14** | 11.79-11.837 | 219.12, 279.03 | HBA derivative 1 |
| **15** | 12.84-12.88 | 219.19, 279.19 | HBA derivative 1 |
| **16** | 13-13.192 | 218.2, 286.95 | HBA derivative 3 |

**
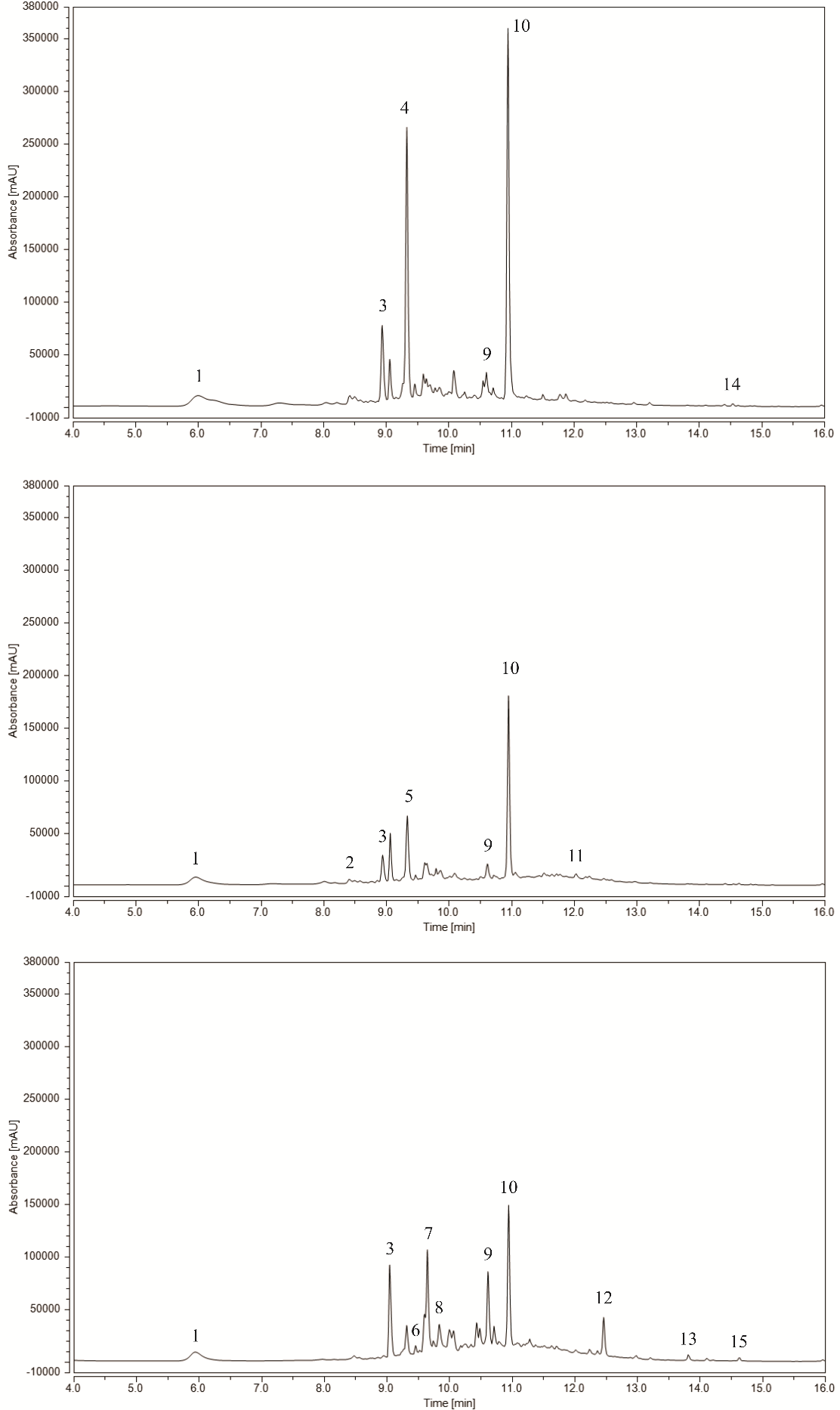
**

S3

S2

S1

**Supplementary Figure 4.** Chromatograms of hydroxycinnamic acids (HBA) detected at 320 nm (free phenolic fraction) by UHPLC-DAD in white *Cabanita* maize at three maturity stages (S1, S2, S3).

**
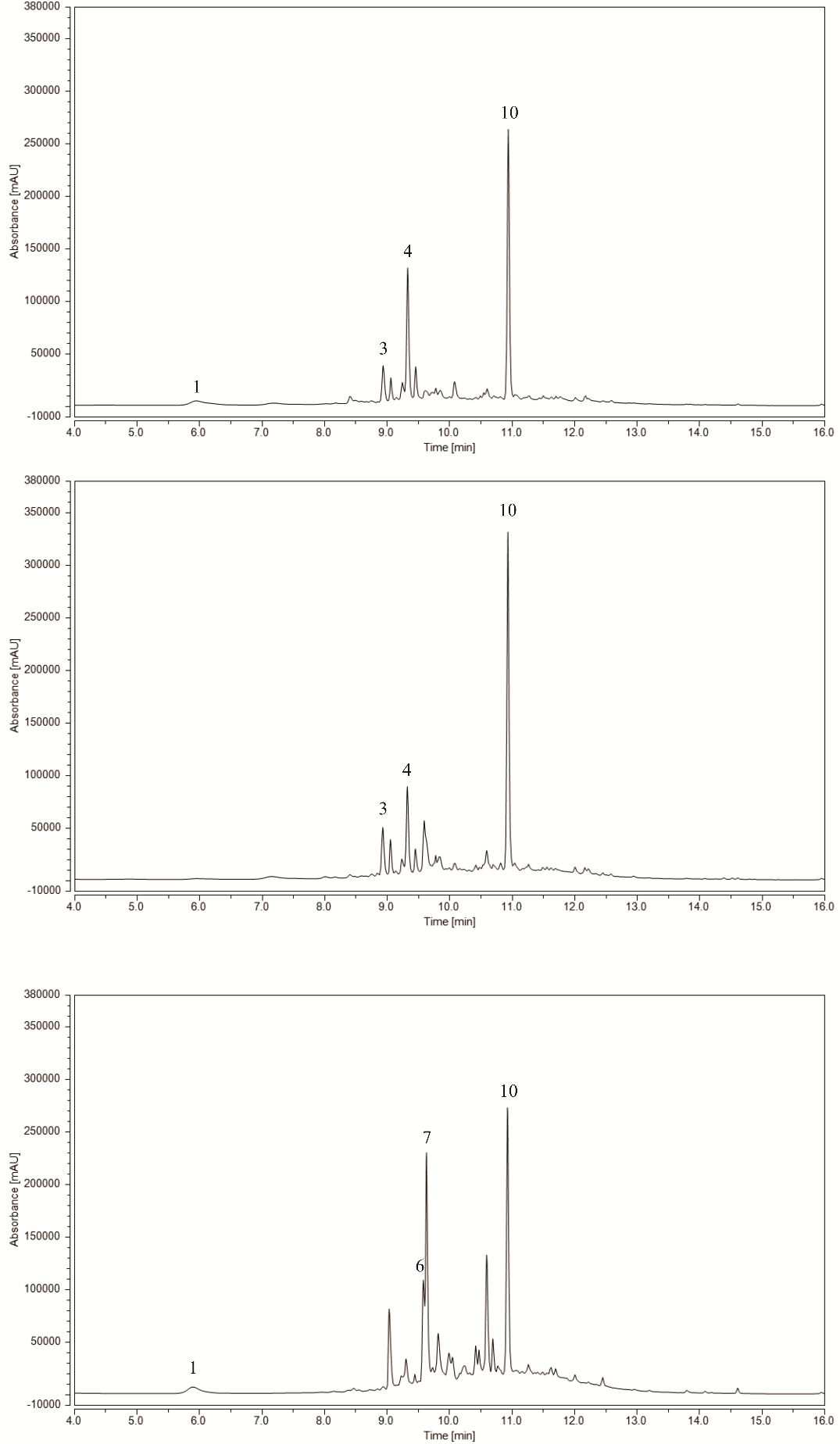
**

S3

S2

S1

**Supplementary Figure 5.** Chromatograms of hydroxycinnamic acids (HBA) detected at 320 nm (free phenolic fraction) by UHPLC-DAD in red *Cabanita* maize at three maturity stages (S1, S2, S3).

**
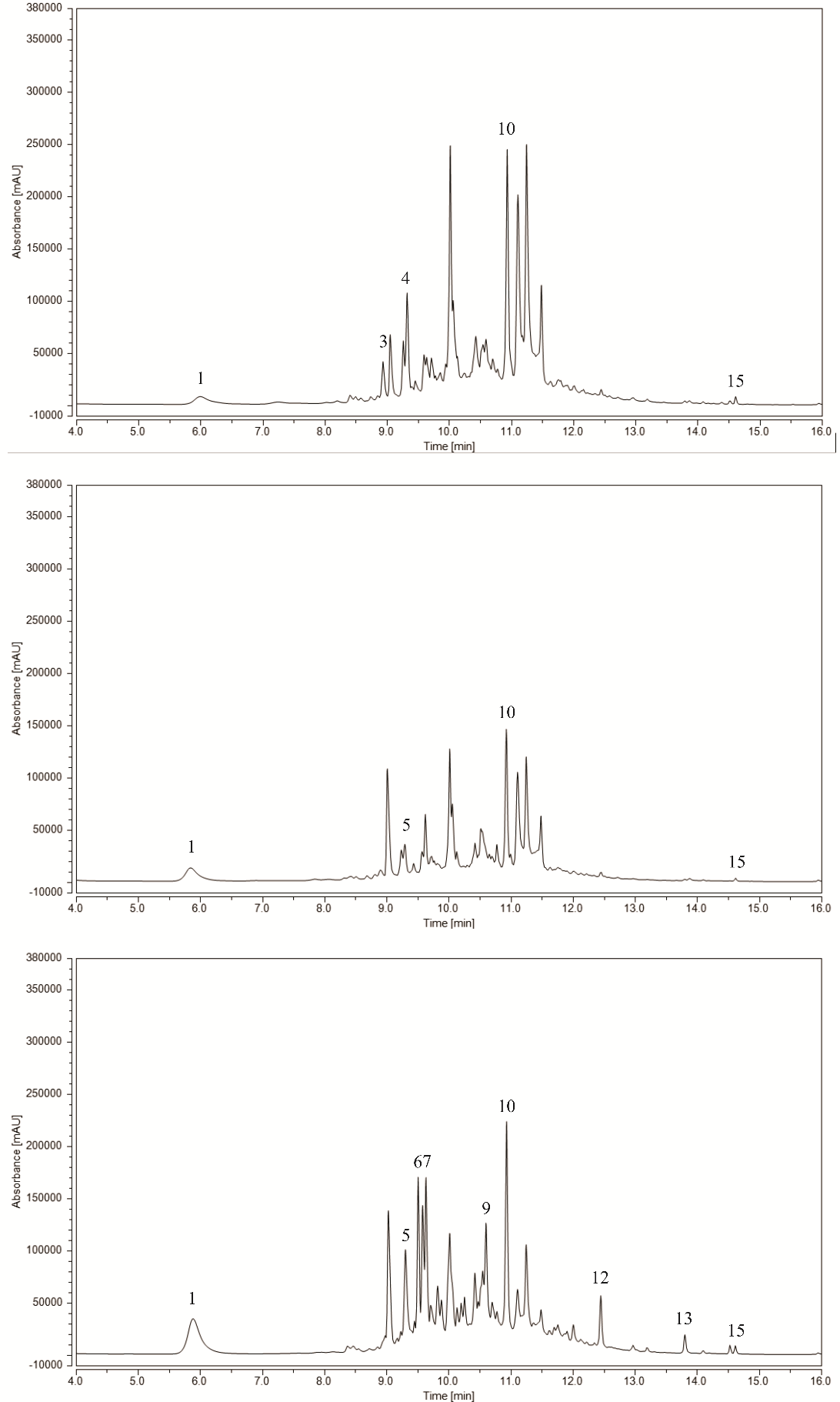
**

S3

S2

S1

**Supplementary Figure 6.** Chromatograms of hydroxycinnamic acids (HBA) detected at 320 nm (free phenolic fraction) by UHPLC-DAD in orange *Cabanita* maize at three maturity stages (S1, S2, S3).

Supplementary Table 4: Retention times, spectra information, and free phenolic compounds (hydroxycinnamic acids, HCA) detected at 320 nm in white, red, and orange Cabanita maize (Supplementary Figures 4-6).

| Nº Peak | Retention time (min) | λ max (nm) | Compound |
| --- | --- | --- | --- |
| **1** | 5.73-6.00 | 205.01, 312.72 | p-coumaric acid derivative |
| **2** | 8.323-8.407 | 292, 316.28 | p-coumaric acid derivative |
| **3** | 8.84-8.938 | 205.56, 310.85 | p-coumaric acid derivative |
| **4** | 9.272-9.328 | 203.37, 322.05 | Ferulic acid derivative |
| **5** | 9.28-9.332 | 202.38, 312.85 | p-coumaric acid derivative |
| **6** | 9.5-9.598 | 205.23, 313.13 | p-coumaric acid derivative |
| **7** | 9.63-9.64 | 223.58, 329.71 | Caffeic acid derivative |
| **8** | 9.792-9.835 | 327.25 | Caffeic acid derivative |
| **9** | 10.59-10.708 | 293.01 | p-coumaric acid derivative |
| **10** | 10.938-10.95 | 217.11, 323.83 | Ferulic acid derivative |
| **11** | 12.022-12.032 | 328.64 | Caffeic acid derivative |
| **12** | 12.44-12.457 | 204.38, 317.4 | Ferulic acid derivative |
| **13** | 13.805-13.81 | 208.32, 328.33 | Caffeic acid derivative |
| **14** | 14.5-14.533 | 216.88, 323.57 | Ferulic acid derivative |
| **15** | 14.6-14.62 | 204.45, 328.37 | Caffeic acid derivative |

**
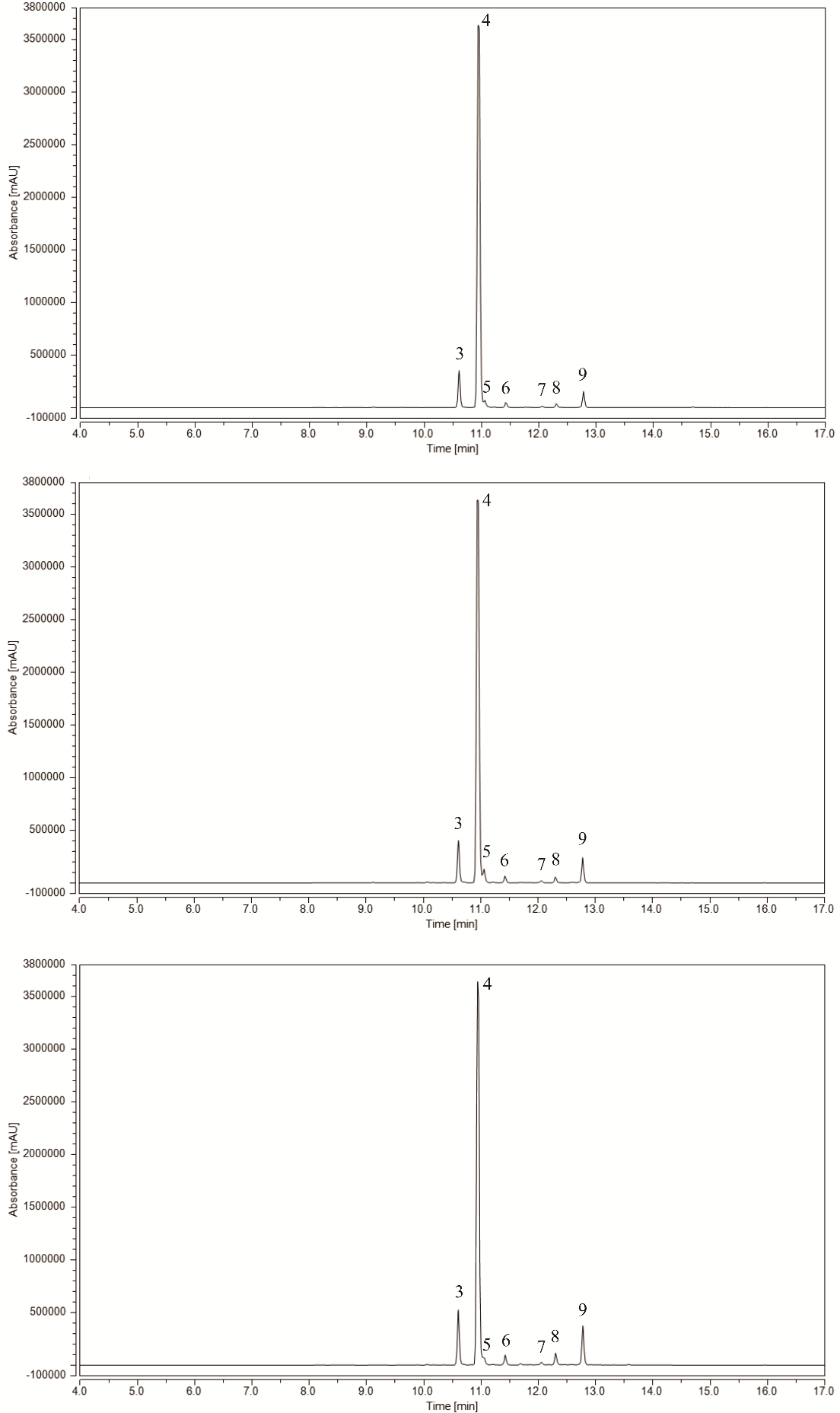
**

S3

S2

S1

**Supplementary Figure 7.** Chromatograms of hydroxycinnamic acids (HBA) detected at 320 nm (bound phenolic fraction) by UHPLC-DAD in white *Cabanita* maize at three maturity stages (S1, S2, S3).

**
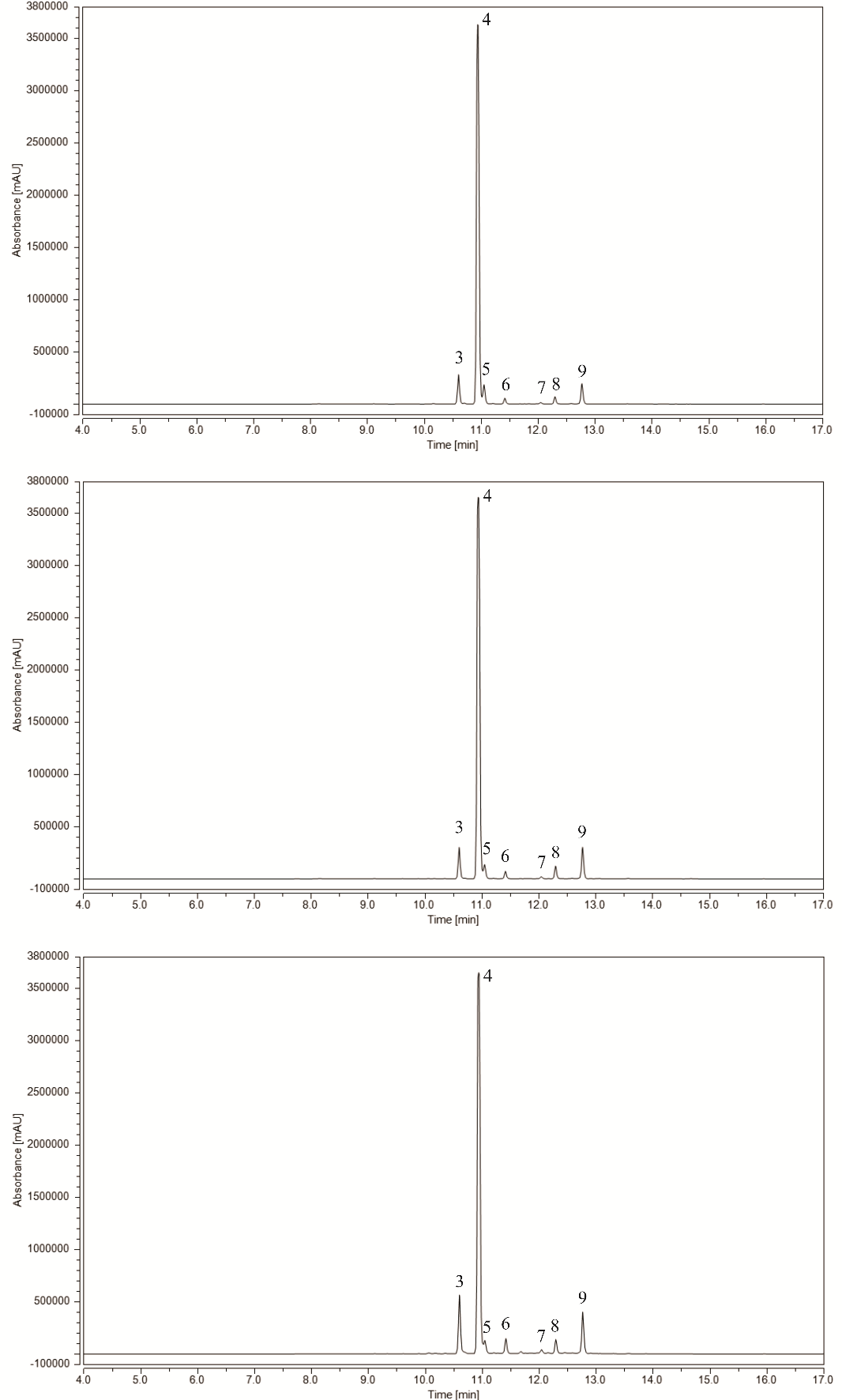
**

S3

S2

S1

**Supplementary Figure 8.** Chromatograms of hydroxycinnamic acids (HBA) detected at 320 nm (bound phenolic fraction) by UHPLC-DAD in red *Cabanita* maize at three maturity stages (S1, S2, S3).

**
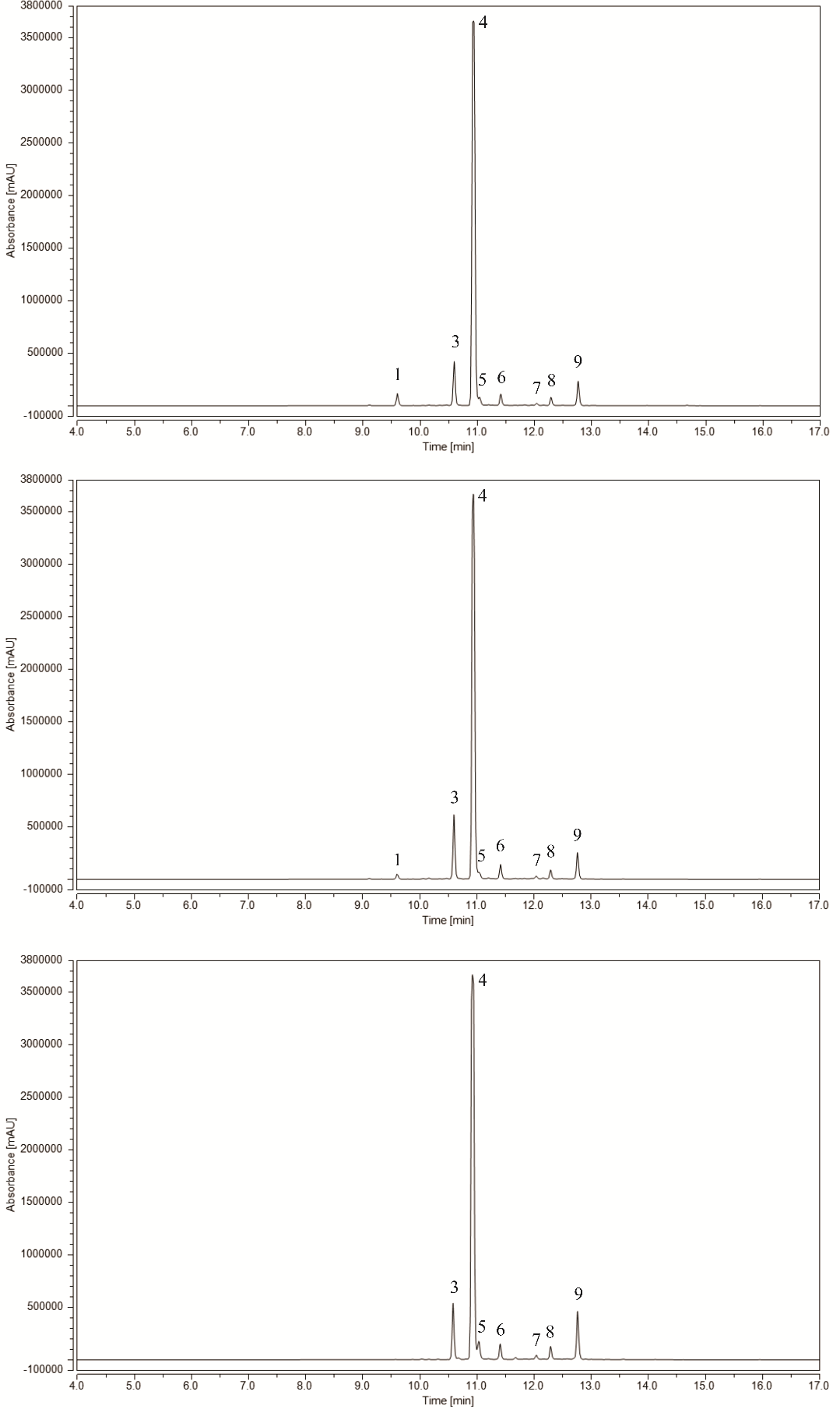
**

S3

S2

S1

**Supplementary Figure 9.** Chromatograms of hydroxycinnamic acids (HBA) detected at 320 nm (bound phenolic fraction) by UHPLC-DAD in orange *Cabanita* maize at three maturity stages (S1, S2, S3).

Supplementary Table 5: Retention times, spectra information, and bound phenolic compounds detected at 320 nm in white, red, and orange *Cabanita* maize (Supplementary Figures 7-9).

| Nº Peak | Retention time (min) | λ max (nm) | Compound |
| --- | --- | --- | --- |
| **1** | 9.56-9.608 | 218.35, 236.96, 323.98 | Ferulic acid derivative |
| **2** | 10.038 | 209.95, 254.89, 326.09 | Ferulic acid derivative |
| **3** | 10.575-10.625 | 225.89, 309.68 | p-coumaric acid |
| **4** | 10.923-10.982 | 217.95, 236.45, 323.07 | Ferulic acid |
| **5** | 11.018-11.087 | 217.91, 319.35 | Ferulic acid derivative |
| **6** | 11.402-11.49 | 219.24, 236.62, 323.34 | Ferulic acid derivative |
| **7** | 12.033-12.295 | 236.52, 321.58 | Ferulic acid derivative |
| **8** | 12.287-12.77 | 218.01, 245.3, 324.39 | Ferulic acid derivative |
| **9** | 12.76-12.887 | 201.42, 324.86 | Ferulic acid derivative |

**
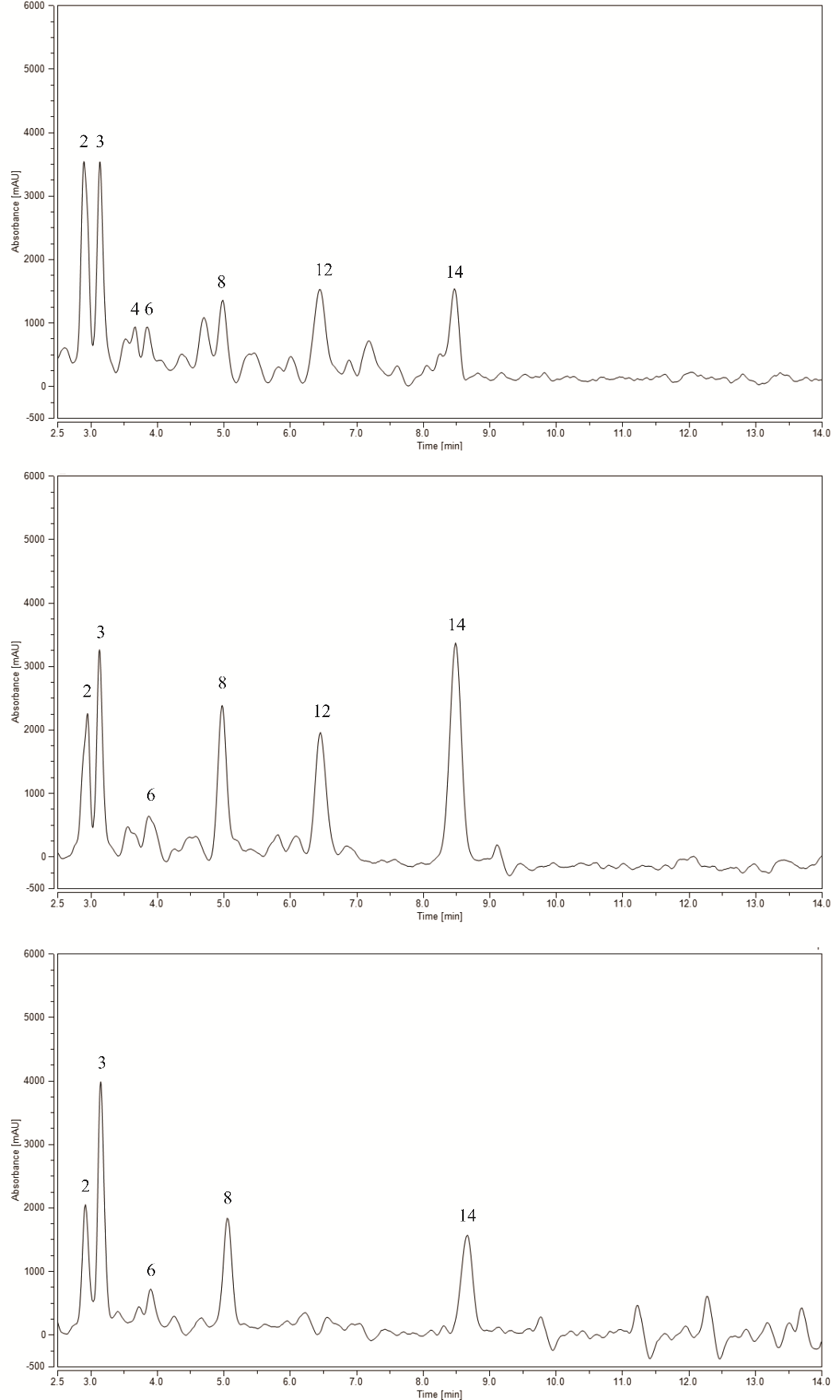
**

S3

S2

S1

**Supplementary Figure 10.** Chromatograms of carotenoid compounds detected at 450 nm by UHPLC-DAD in white *Cabanita* maize at three maturity stages (S1, S2, S3).


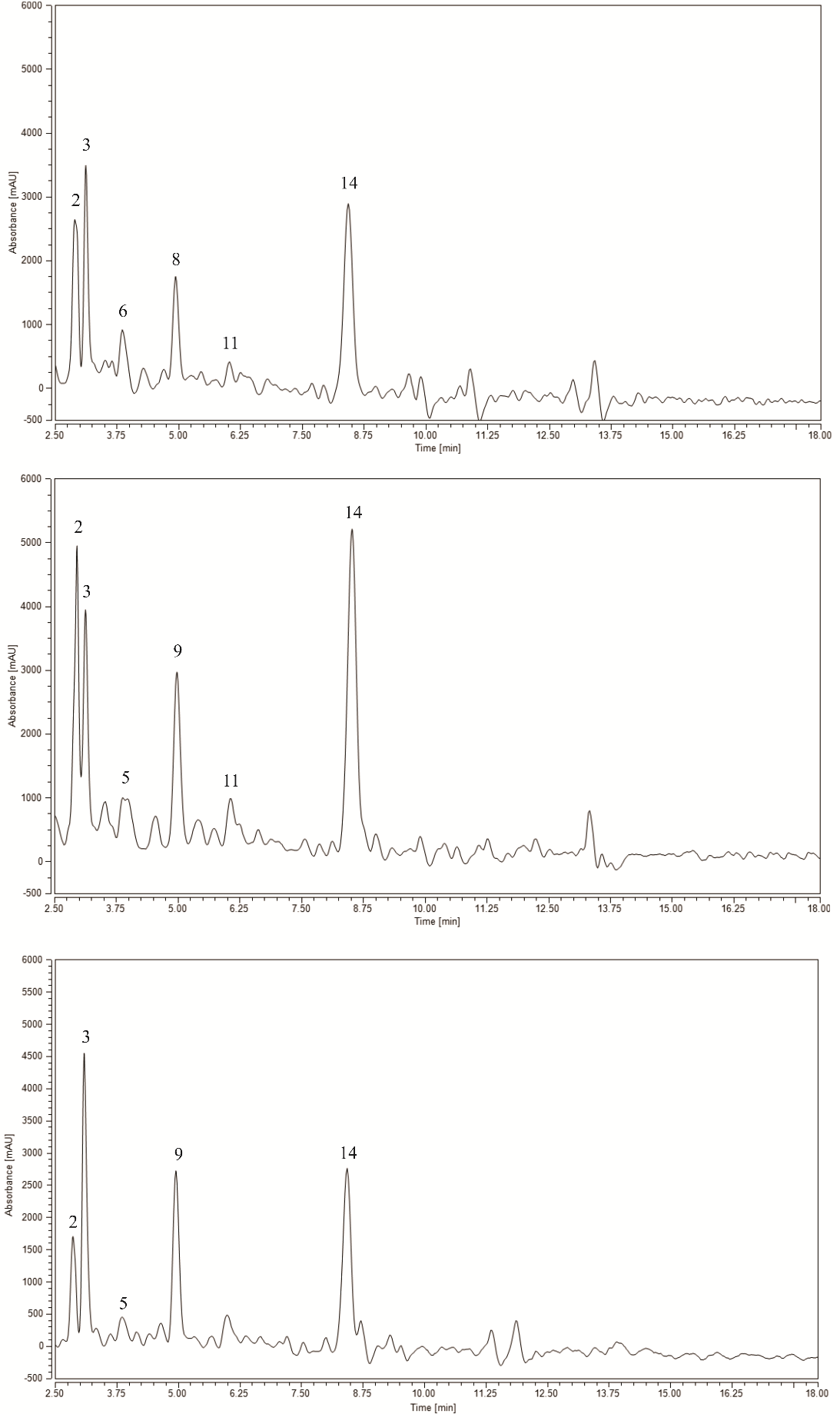


S3

S2

S1

**Supplementary Figure 11.** Chromatograms of carotenoid compounds detected at 450 nm by UHPLC-DAD in red *Cabanita* maize at three maturity stages (S1, S2, S3).


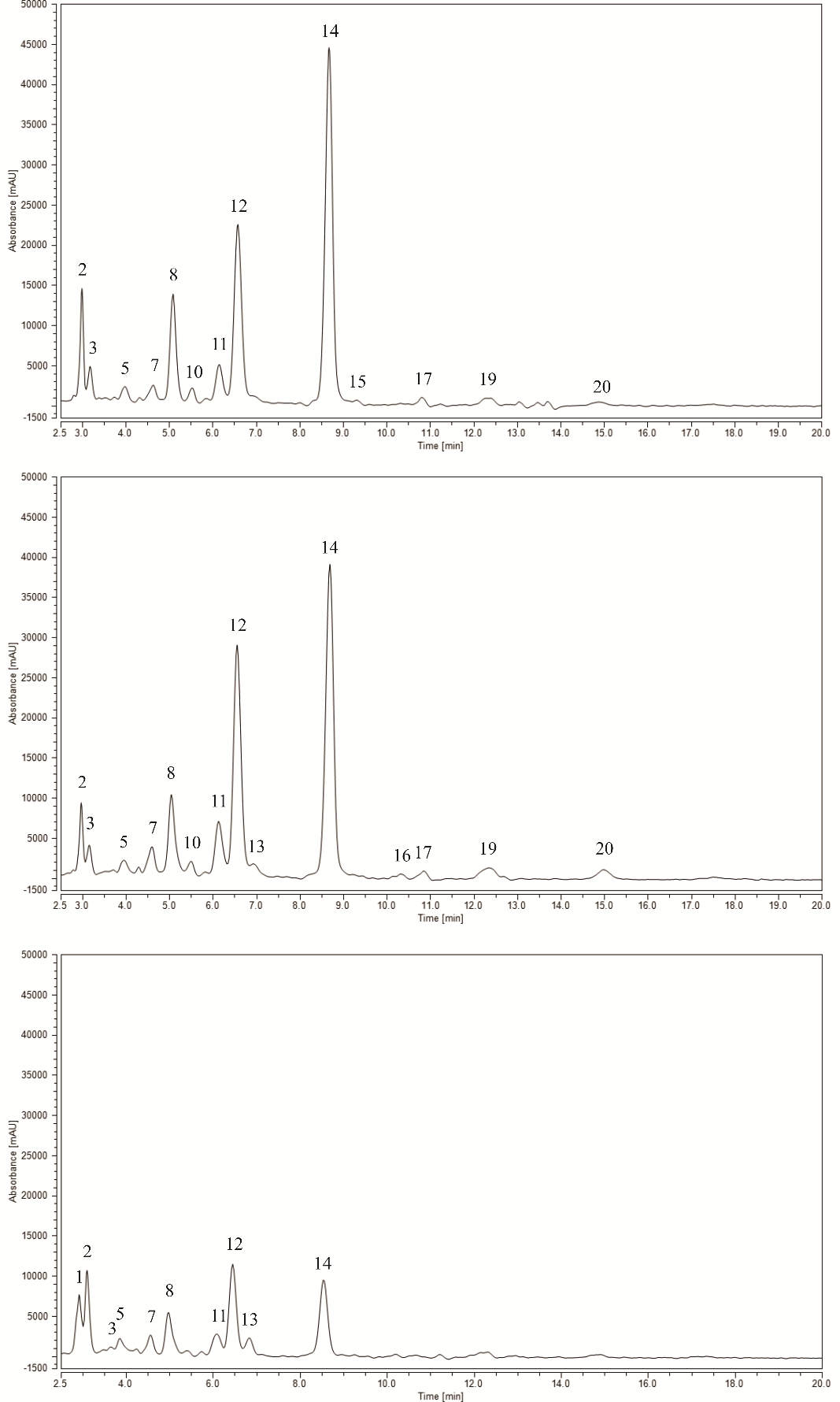


S3

S2

S1

**Supplementary Figure 12.** Chromatograms of carotenoid compounds detected at 450 nm by UHPLC-DAD in orange *Cabanita* maize at three maturity stages (S1, S2, S3).

Supplementary Table 6: Retention times, spectra information, and carotenoid compounds detected at 450 nm in white, red, and orange *Cabanita* maize (Supplementary Figures 10-12).

| Nº Peak | Retention time (min) | λ max (nm) | Compound |
| --- | --- | --- | --- |
| **1** | 2.853-2.99 | 468.95, 440.43, 416.85 | Neoxanthin isomer (~13-cis-neoxanthin) |
| **2** | 3.063-3.177 | 469.15; 440.62, 417.06 | All-trans-neoxanthin |
| **3** | 3.623-3.7 | 468.6, 439.29 | Unidentified 1 |
| **4** | 3.837-3.977 | 464.3, 435.88, 412.22 | Violaxanthin isomer (~9-cis-violaxanthin) |
| **5** | 3.823-3.973 | 466.92, 442.08 | Violaxanthin isomer (~9-cis-violaxanthin) |
| **6** | 4.5-4.63 | 448.18, 421.71, 398.51 | Unidentified 2 |
| **7** | 4.917-5.083 | 465.56, 439.3 | Lutein isomer (~13 cis-lutein) |
| **8** | 4.927-5.013 | 466.21, 440.56 | Lutein isomer (~13 cis-lutein) |
| **9** | 5.36-5.523 | 471.04 | Unidentified 3 |
| **10** | 5.98-6.147 | 474.75, 448.33 | Zeaxanthin isomer (~13 cis-zeaxanthin) |
| **11** | 6.34-6.573 | 468.72, 443.73 | All-trans-lutein |
| **12** | 6.797-6.983 | 472.49, 444.55 | Unidentified 4 |
| **13** | 8.287-8.683 | 452.97, 427.81 | All-trans-zeaxanthin |
| **14** | 9.12-9.303 | 477.02, 450.54 | Lutein isomer (~9 or 9' cis lutein) |
| **15** | 10.107-10.317 | 466.28, 441.53 | Lutein isomer (~9 or 9' cis lutein) |
| **16** | 10.597-10.84 | 467.8, 440.33, 415.57 | Cryptoxanthin isomer (~13 cis-β-cryptoxanthin) |
| **17** | 11.637-11.987 | 472.81, 442.44, 422.57 | Cryptoxanthin isomer (~13 cis-β-cryptoxanthin) |
| **18** | 12.133-12.34 | 469.91, 444.99 | Cryptoxanthin isomer (~13 cis-β-cryptoxanthin) |
| **19** | 14.303-14.46 | 471.04, 445.46 | All-trans-β-cryptoxanthin |
| **20** | 14.567-14.973 | 477.1, 450.73 | Cryptoxanthin isomer (~9 or 9'-cis-β-cryptoxanthin) |
